# Regulation of carcinogenesis and mediation through Wnt/β-catenin signaling by 3,3′-diindolylmethane in an enzalutamide-resistant prostate cancer cell line

**DOI:** 10.1038/s41598-020-80519-3

**Published:** 2021-01-13

**Authors:** Chih-Wei Tsao, Jia-Sin Li, Ya-Wen Lin, Sheng-Tang Wu, Tai-Lung Cha, Chin-Yu Liu

**Affiliations:** 1 Division of Urology, Department of Surgery, Tri-Service General Hospital, National Defense Medical Center, Taipei, Taiwan; 2 Department of Nutritional Science, Fu Jen Catholic University, No. 510, Zhongzheng Rd., Xinzhuang Dist., New Taipei City, 24205 Taiwan; 3 Department of Microbiology and Immunology, National Defense Medical Center, Taipei, Taiwan

**Keywords:** Prostate cancer, Urology, Cancer, Cancer therapy, Urological cancer, Nutrition, Cell growth, Cell migration, Cell signalling

## Abstract

Enzalutamide (ENZ) is an important drug used to treat castration-resistant prostate cancer (CRPC), which inhibits androgen receptor (AR) signaling. Previous study showed that 3,3′-diindolylmethane (DIM) is an AR antagonist that also inhibits Wnt signaling and epithelial-mesenchymal transition (EMT). To investigate whether combined treatment with ENZ and DIM can overcome ENZ resistance by regulating Wnt signaling to inhibit AR signaling and EMT in ENZ-resistant prostate cancer cells, 22Rv1 cells were cultured in normal medium and treated with ENZ, DIM, and DIM with ENZ. Exposure of ENZ-resistant cells to both DIM and ENZ significantly inhibited cell proliferation without cytotoxicity and invasion in comparison with the control. DIM significantly increased the E-cadherin expression and inhibited the expressions of Vimentin and Fibronectin, subsequently inhibiting EMT. Co-treatment with ENZ and DIM significantly increased the expressions of GSK3β and APC and decreased the β-catenin protein expression, causing inhibition of Wnt signaling and AR expression, it also significantly decreased the AR-v7 expression and down-regulated AR signaling. Via suppression of Wnt and AR signaling, co-treatment increased the E-cadherin and decreased the Vimentin and Fibronectin RNA and protein expressions, then inhibited EMT. Co-treatment with DIM and ENZ regulated Wnt signaling to reduce not only the AR expression, but also the AR-v7 expression, indicating suppression of EMT that inhibits cancer cell proliferation, invasion and migration to ameliorate ENZ resistance.

## Introduction

Prostate cancer (PCa) is not only the second most common cancer in men; it is also the fifth leading cause of cancer mortality among men worldwide^1^. In Taiwan, the prevalence and mortality of PCa are increasing, and around 50% of patients are diagnosed with PCa at the late stage^2^. At the late stage, androgen deprivation therapy (ADT) is considered the standard therapy^3^; however, after the beginning of ADT, the duration of response to castration is short and, in almost all patients, is followed by the emergence of a castration-resistant phenotype^4^. Recurrence of PCa is regarded as castration-resistant prostate cancer (CRPC). It has been confirmed that CRPC is still an androgen-dependent disease that relies on androgen receptor (AR) signaling^5^. AR signaling can be activated through various mechanisms, such as AR overexpression, binding of alternate AR ligands, upregulation of AR coactivators and downregulation of AR corepressors, steroidogenesis, inflammation pathways, growth-factor signaling, and Wnt/β-catenin signaling^6^. Therefore, second-generation anti-androgen drugs, such as abiraterone and enzalutamide (ENZ), were developed as new therapies for CRPC^7^.

ENZ, also named MDV3100, is an AR antagonist that competitively antagonizes androgen binding to AR and inhibits nuclear translocation of AR, recruitment of AR co-factors, and AR binding to DNA^8^. ENZ has been proven to increase the overall survival duration and improve quality of life of PCa patients. Owing to its safety characteristics, it has become a critical drug for use at different stages of PCa^9^. Although ENZ has many benefits, 20–40% of patients have primary resistance, and nearly all treated CRPC patients develop ENZ resistance eventually^10^. Recent evidence has shown that several mechanisms are involved in ENZ resistance, including AR mutation, the existence of AR splice variants (AR-Vs), AR and glucocorticoid receptor (GR) overexpression, autophagy, intracrine androgen biosynthesis, and epithelial-mesenchymal transition (EMT)^11–13^.

Wnt proteins are cysteine-rich, secreted lipoglycoproteins that play critical roles in normal embryonic development, and are involved in a variety of biological processes, but aberrant activation of the Wnt signaling pathway has been noted in carcinogenesis^14^. Our previous studies have shown that dysregulation of Wnt signaling is associated with hepatoma^15^, ovarian cancer^16,17^, and cervical cancer^18^, while other studies have demonstrated its involvement in prostate cancer^19^ and colorectal cancer^20^ in terms of affecting cell proliferation, invasion or migration. The two distinct pathways of Wnt signals are a canonical or Wnt/β‐catenin pathway and a noncanonical pathway or pathways that are β-catenin-independent^21^. The stabilization and nuclear translocation of β-catenin play crucial roles while Wnt signaling is engaged. β-catenin no longer binds to some proteins, such as adenomatous polyposis coli (APC) protein and glycogen synthase kinase‑3 (GSK3), but does bind to T-cell factor (TCF) family transcription factors, whereupon it activates target genes determining carcinogenesis, including *MYC*, *MMP7*, *VEGF* and *AR*^14,19^.

Relatively high gene expression levels of both β-catenin and AR have been observed in CRPC^22^. Of several mechanisms involved in ENZ-resistant CRPC, aberrant AR signaling is a crucial one. In general, AR signaling is activated while AR is bound to androgen, and then translocates to the nucleus to bind with androgen-responsive elements (ARE) in the promoter regions of target genes^6^. AR-Vs have been found to translocate into the nucleus without androgen, and can form homodimers or heterodimers with androgen-bound AR^23^. AR-Vs may be associated with the initiation and progression of PCa. Evidence has shown that AR-V expressions are involved in EMT, which plays important roles in cancer progression and drug resistance^23–25^. Among approximately 20 different variants, increased AR-v7 expression has been identified in CRPC patients, and is related to ENZ resistance^26,27^. Moreover, interaction of AR with Wnt signaling has been suggested, and both could activate the process of EMT^24,28,29^. Therefore, a new adjuvant therapy might be helpful in improving the treatment of ENZ-resistant CRPC.

According to epidemic evidence, high intakes of cruciferous vegetables reduce the risk of PCa, especially in the early stages^30–32^, and dietary and plant-based drugs have been suggested as possible alternative strategies in the treatment of cancer. The phytochemical indole-3-carbinol (I3C) and its metabolite 3,3′-diindolylmethane (DIM) are present in high levels in cruciferous vegetables, and have been shown to exert anticancer effects, but DIM has a greater bioactivity and is safer than I3C^33,34^. Previous studies have shown that DIM induces cell-cycle arrest in AR positive, p53 wild-type LNCaP and AR negative, p53 mutant DU145 human prostate cancer cell lines^35^. It also has inhibitory effects on AR signaling, Wnt signaling and the process of EMT^30,33,34,36^. Moreover, in clinical experiments, a formulated DIM (Bio-Response 3,3′-diindolylmethane, BR-DIM) was used as an adjuvant therapy in the early stages of PCa, and a good response was observed^29,30,34^. However, no research has been performed to examine the effects of DIM through Wnt/β-catenin signaling in the late stages of ENZ-resistant PCa. Therefore, in the current study, we focused on AR, AR-v7 and Wnt signaling to investigate whether combined treatment with ENZ and DIM could overcome ENZ resistance by regulating Wnt signaling to inhibit AR signaling and EMT in ENZ-resistant PCa cells. We found that co-treatment with DIM and ENZ inhibited prostate cancer cell proliferation, invasion and migration through regulating Wnt signaling to reduce the expressions of AR and AR-v7.

## Results

### DIM and ENZ treatment suppress cell proliferation and colony formation of PCa cells

**Figure 1:**
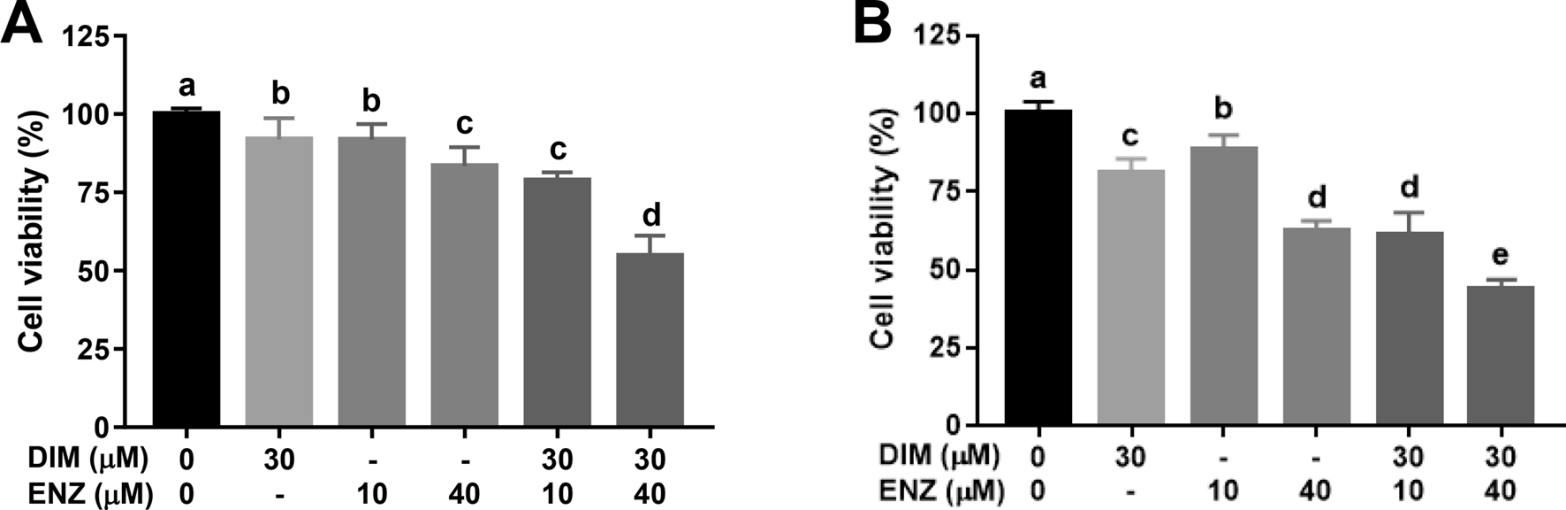
Inhibition of cell proliferation by DIM (30 μM) and ENZ (10 or 40 μM), alone and in combination, in 22Rv1 cells at 48 h (**A**) and 72 h (**B**) post-exposure (n = 6). *DIM* 3,3′-diindolylmethane; *ENZ* enzalutamide. Values are presented as means ± SD. ^a,b,c,d^Bars without the same letters at the top indicate statistically-significant differences between treatments when compared with each other (one-way ANOVA, Duncan’s new multiple range test, *p* < 0.05).

Exposure to a low dose of ENZ for 48 and 72 h had no suppressive effect on 22Rv1 cells (data not shown). It was confirmed that 22Rv1 cells exhibited ENZ resistance. Treatment of cells with DIM alone for 48 and 72 h caused significant dose- and time-dependent inhibition of cell proliferation, with 25% suppression following 72 h of exposure to 30 μM DIM. Therefore, in order to investigate the adjuvant effects of DIM on ENZ resistance, 22Rv1 cells were treated with DIM and ENZ simultaneously, and the results showed that treatment of 22Rv1 cells with 30 μM DIM alone for 48 and 72 h significantly decreased the cell viability (Fig. 1; *p* < 0.05 vs. control), and the effect of co-treatment with DIM and 10 μM ENZ was similar to that of treatment with 40 μM ENZ. Co-exposure to DIM and ENZ resulted in significant inhibition of cell proliferation (Fig. 1; *p* < 0.05 vs. DIM or ENZ alone).

**Figure 2:**
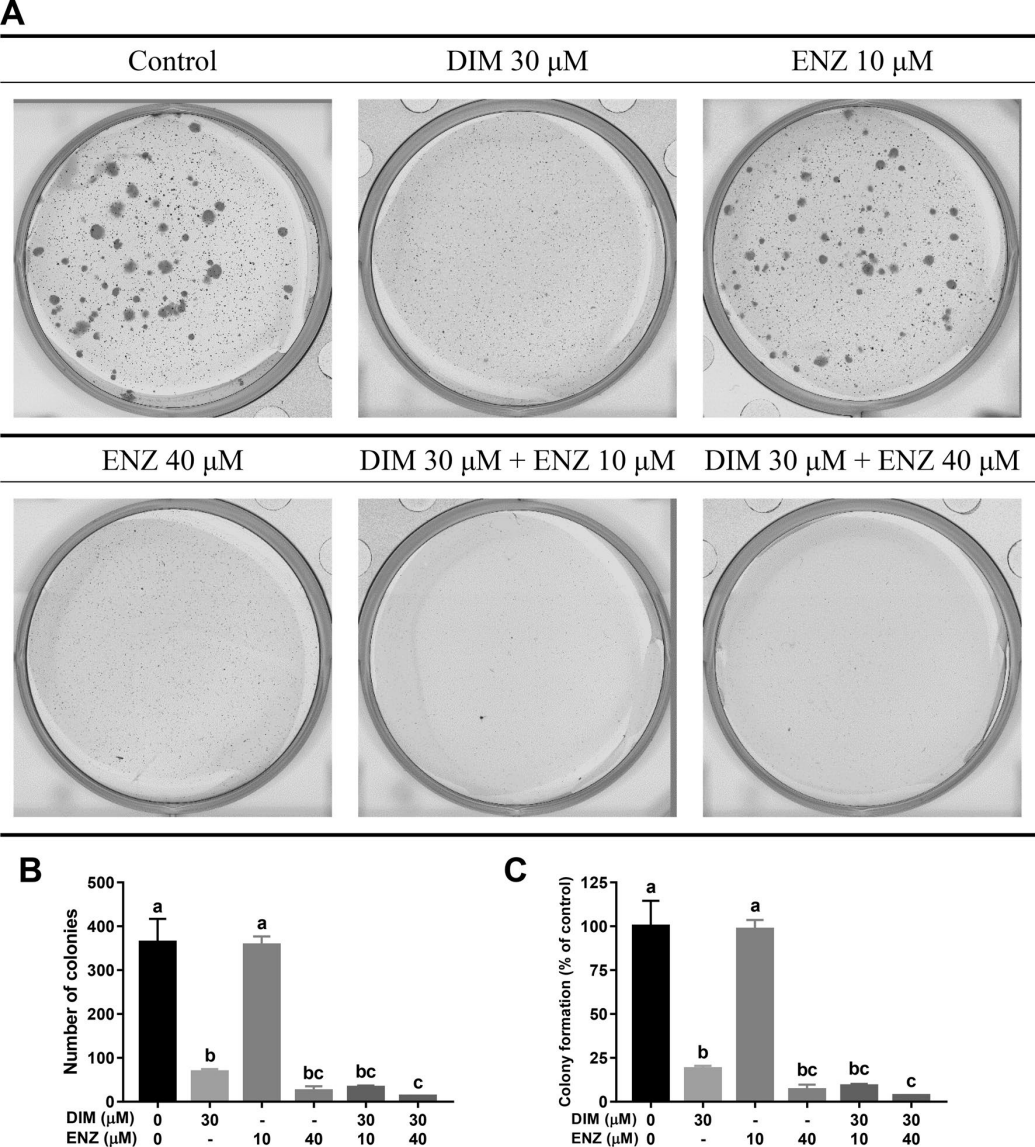
DIM and ENZ treatment suppress cell colony formation of PCa cells (n = 3). *DIM* 3,3′-diindolylmethane; *ENZ* enzalutamide. Representative images (**A**), number of colonies (**B**), colony formation normalized to control (**C**). Values are presented as means ± SD. ^a,b,c^Bars without the same letters at the top indicate statistically-significant differences between treatments when compared with each other (one-way ANOVA, Duncan’s new multiple range test, *p* < 0.05).

In 22Rv1 cells, treatment with DIM alone significantly inhibited the clonogenic ability (Fig. 2; *p* < 0.05 vs. control), while there was no suppression when cells were treated with 10 μM ENZ. In cells treated with a high concentration of ENZ, colony formation was significantly inhibited (Fig. 2; *p* < 0.05 vs. control), and the inhibition was of the same magnitude as that caused by treatment with 30 μM DIM. Combined DIM and ENZ treatment significantly increased the inhibition of colony formation (Fig. 2; *p* < 0.05 vs. control and 10 μM ENZ). The results of the present study indicated that ENZ and DIM may have a synergistic effect in suppressing cell proliferation and colony formation in PCa cells.

### DIM potentiates the inhibition effect of ENZ on PCa cell invasion

**Figure 3:**
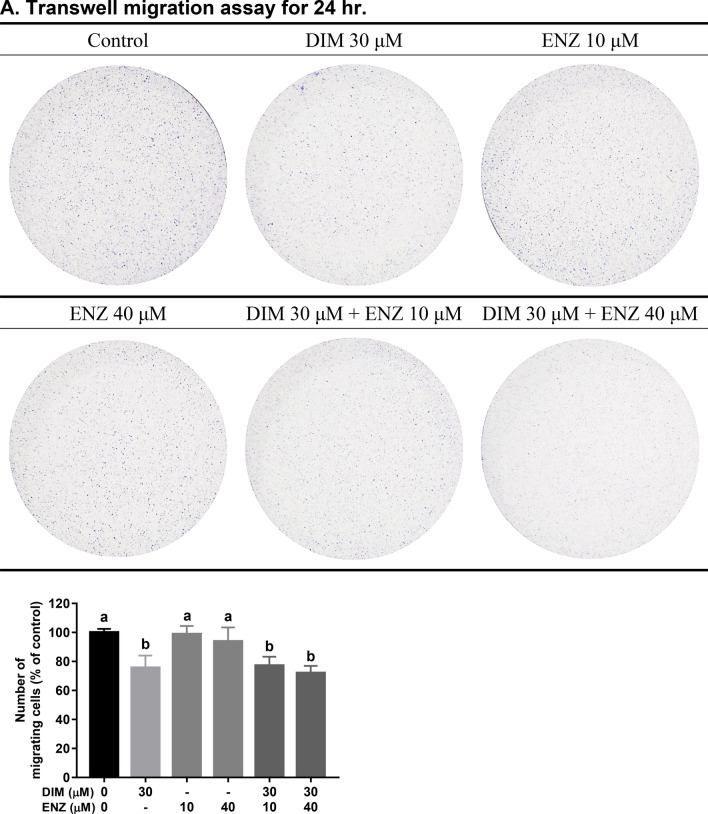

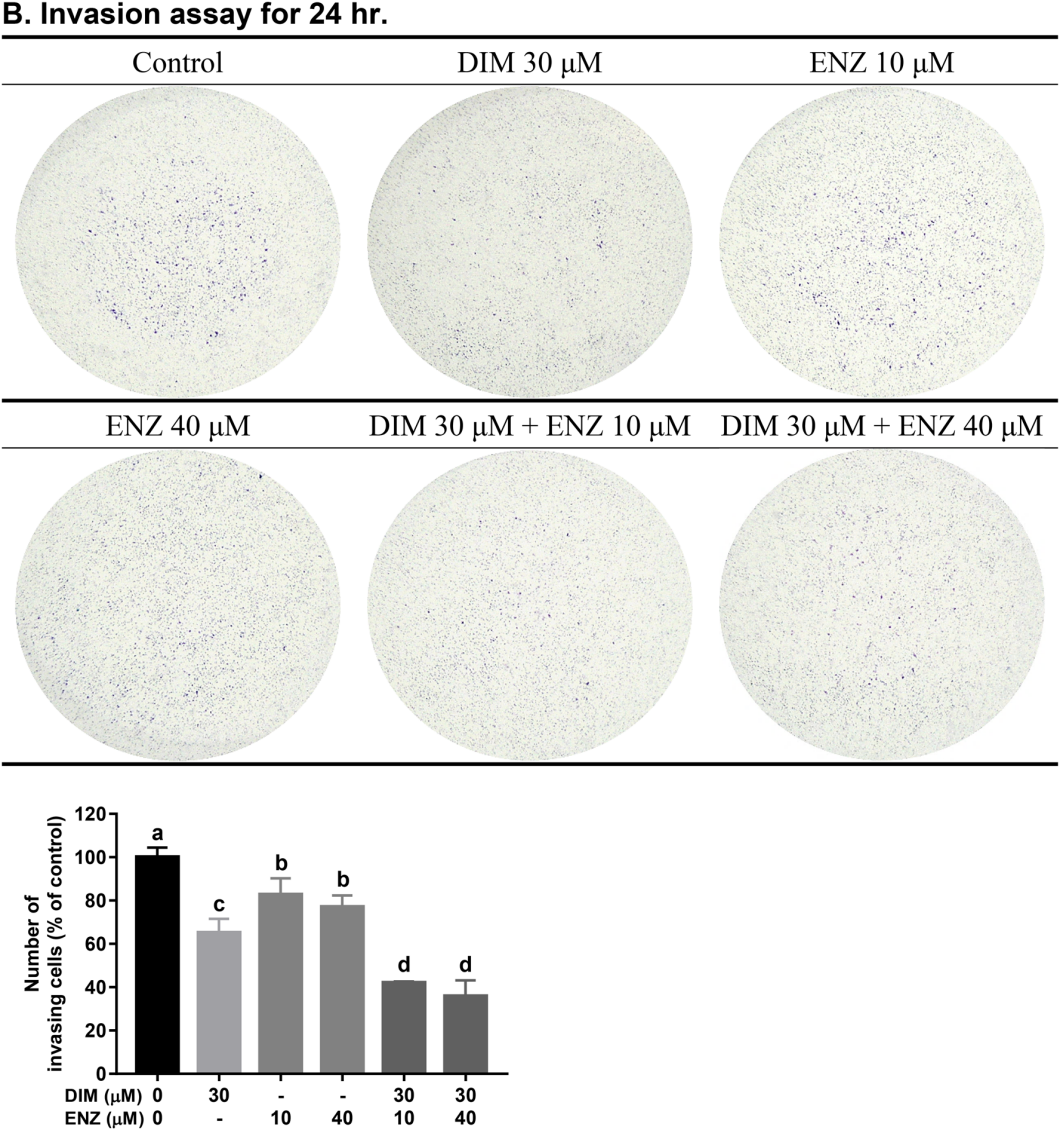
DIM potentiates the inhibition effect of ENZ on PCa cell invasion (n = 3). *DIM* 3,3′-diindolylmethane; *ENZ* enzalutamide. Results of cell migration (**A**) and invasion (**B**) assays. Values are presented as means ± SD. ^a,b,c^Bars without the same letters at the top indicate statistically-significant differences between treatments when compared with each other (one-way ANOVA, Duncan’s new multiple range test, *p* < 0.05).

In order to examine whether combined treatment with DIM and ENZ resulted in stronger inhibition of invasion in PCa cells, 22Rv1 cells were treated with DIM and ENZ alone and together for 24 h. The results illustrated that both DIM and ENZ significantly inhibited cell invasion (Fig. 3B; *p* < 0.05 vs. control), but DIM had a better inhibition effect (Fig. 3B; *p* < 0.05 vs. 10 and 40 μM ENZ). In addition, exposure to DIM and ENZ in combination significantly increased the efficacy of ENZ in suppressing cell invasion (Fig. 3B; *p* < 0.05 vs. 10 and 40 μM ENZ). The results of the present study indicated that DIM may potentiate the efficacy of ENZ with regards to invasion of 22Rv1 cells.

### DIM and ENZ co-treatment augments the suppression of PCa cell migration

**Figure 4:**
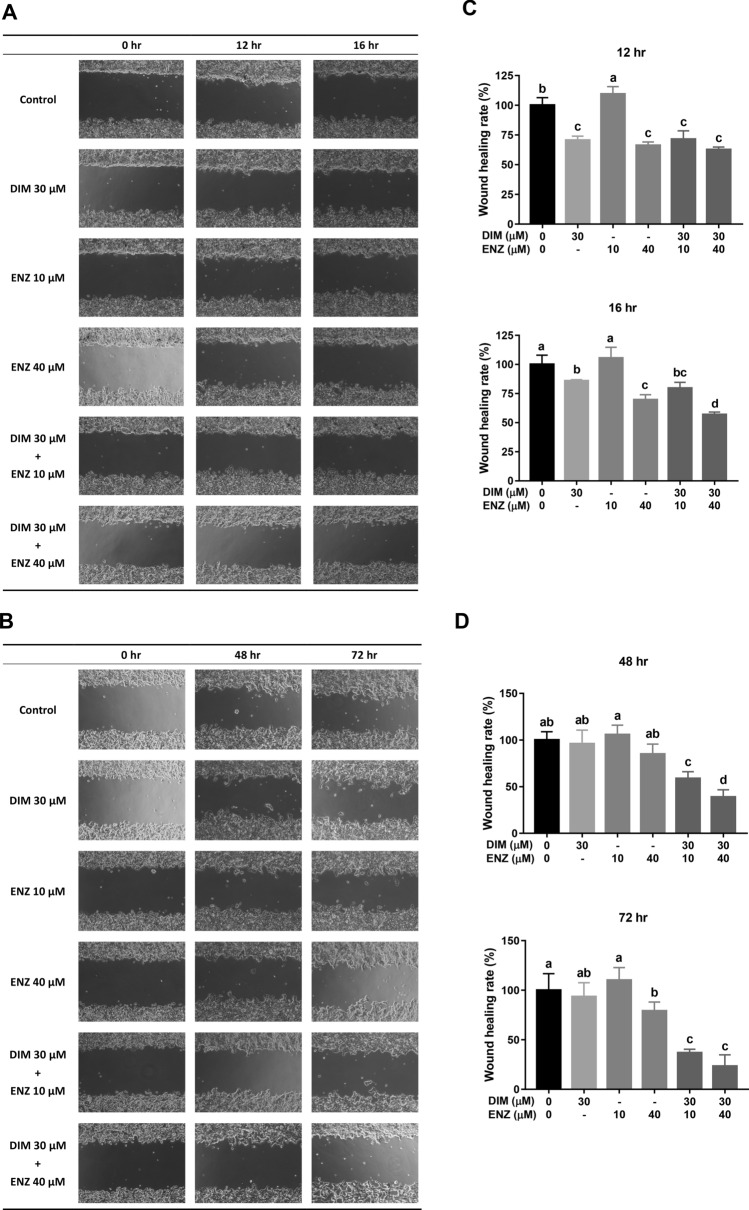
DIM and ENZ co-treatment augments the suppression of PCa cell migration (n = 4). Representative images at 12 and 16 h (**A**), 48 and 72 h (**B**); wound-healing rate (**C**). *DIM* 3,3′-diindolylmethane; *ENZ* enzalutamide. Values are presented as means ± SD. ^a,b,c^Bars without the same letters at the top indicate statistically-significant differences between treatments when compared with each other (one-way ANOVA, Duncan’s new multiple range test, *p* < 0.05).

To investigate whether combined treatment with DIM and ENZ inhibited PCa cell migration, a wound-healing assay and a Transwell migration assay were employed. As shown in Fig. 4A,C, treatment with 10 μM ENZ did not inhibit cancer cell migration; however, exposure to DIM significantly suppressed cell migration (*p* < 0.05 vs. control and 10 μM ENZ). Treatment with ENZ (10 and 40 μM) and DIM combined resulted in a stronger inhibition effect (*p* < 0.05 vs. control and 10 μM ENZ). Furthermore, as shown in Fig. 3A, treatment of 22Rv1 cells with ENZ alone did not repress cell migration, but treatment with DIM or combined treatment with DIM and ENZ significantly inhibited cancer cell migration (*p* < 0.05 vs. control and ENZ alone). The results showed that combined treatment with DIM and ENZ resulted in better suppression of migration in PCa cells after 24 h. Therefore, a wound-healing assay was employed to detect cell migration at 48 and 72 h. Exposure to DIM and ENZ alone did not suppress migration of 22Rv1 cells (Fig. 4B,D), but combined treatment with DIM and ENZ significantly suppressed migration of 22Rv1 cells (Fig. 4B,D; *p* < 0.05 vs. control, DIM and ENZ alone). The results of the present study indicated that co-treatment with DIM and ENZ resulted in better suppression of migration of PCa cells.

### DIM and ENZ co-treatment inhibits Wnt signaling in PCa cells

**Figure 5:**
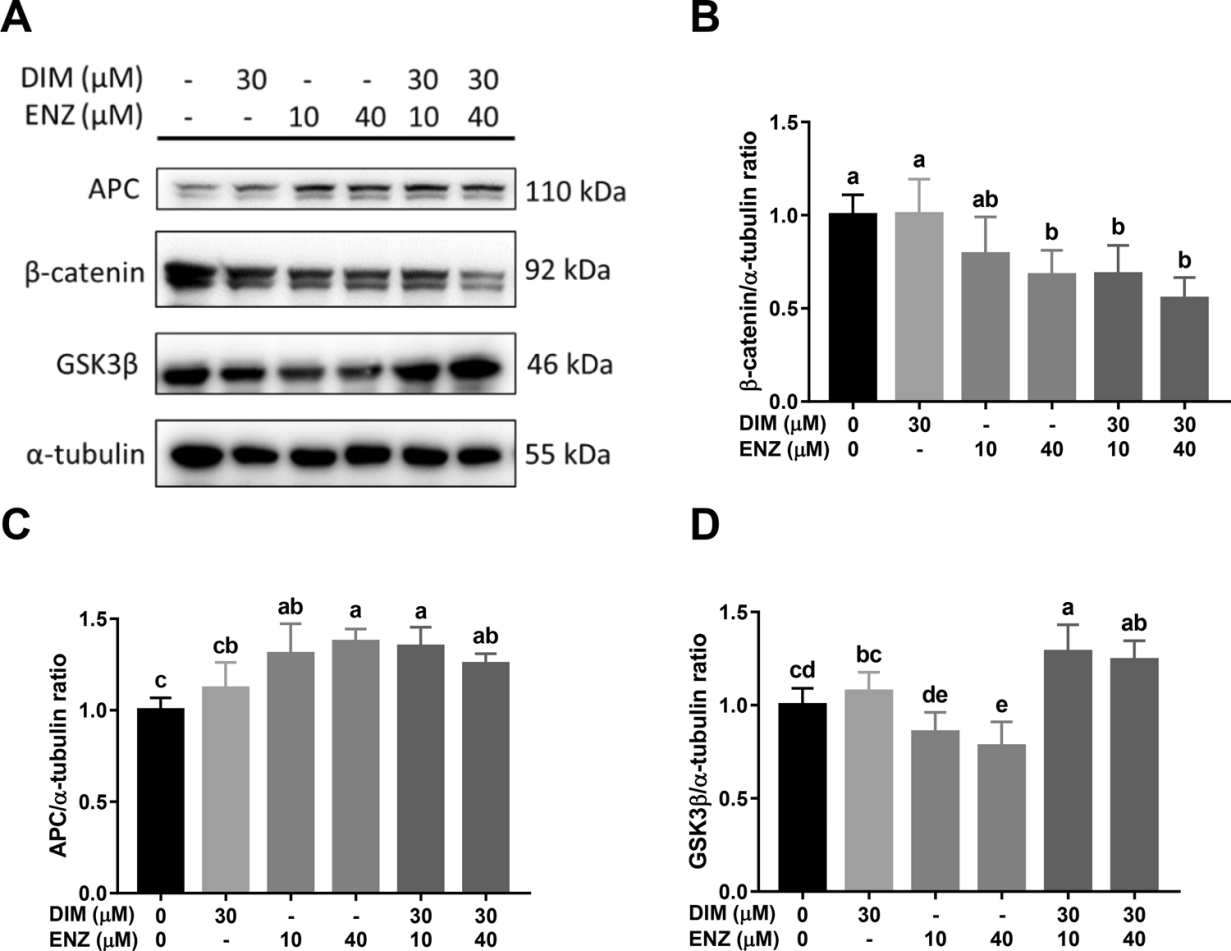
Inhibition of Wnt signaling by DIM and ENZ co-treatment in 22Rv1 cells at 48 h. Representative western blot image (**A**); quantitative results for β-catenin (**B**), APC (**C**), and GSK3β (**D**). *DIM* 3,3′-diindolylmethane; *ENZ* enzalutamide. Values are presented as means ± SD. ^a,b,c,d^Bars without the same letters at the top indicate statistically-significant differences between treatments when compared with each other (one-way ANOVA, Duncan’s new multiple range test, *p* < 0.05).

Wnt signaling is an important mechanism in CRPC. In order to elucidate whether co-treatment with DIM and ENZ inhibits Wnt signaling, western blot analysis was employed to detect the expressions of β-catenin, APC, and GSK3β, which play important roles in Wnt signaling. After 48 h of treatment, exposure to DIM and ENZ in combination significantly increased the expressions of APC and GSK3β, and downregulated the expression of β-catenin (Fig. 5; *p* < 0.05 vs. control). Compared with treatment of 22Rv1 cells with 10 μM ENZ alone, co-treatment with ENZ and DIM significantly upregulated the level of GSK3β and decreased the expression of β-catenin (Fig. 5; *p* < 0.05 vs. 10 μM ENZ).

**Figure 6:**
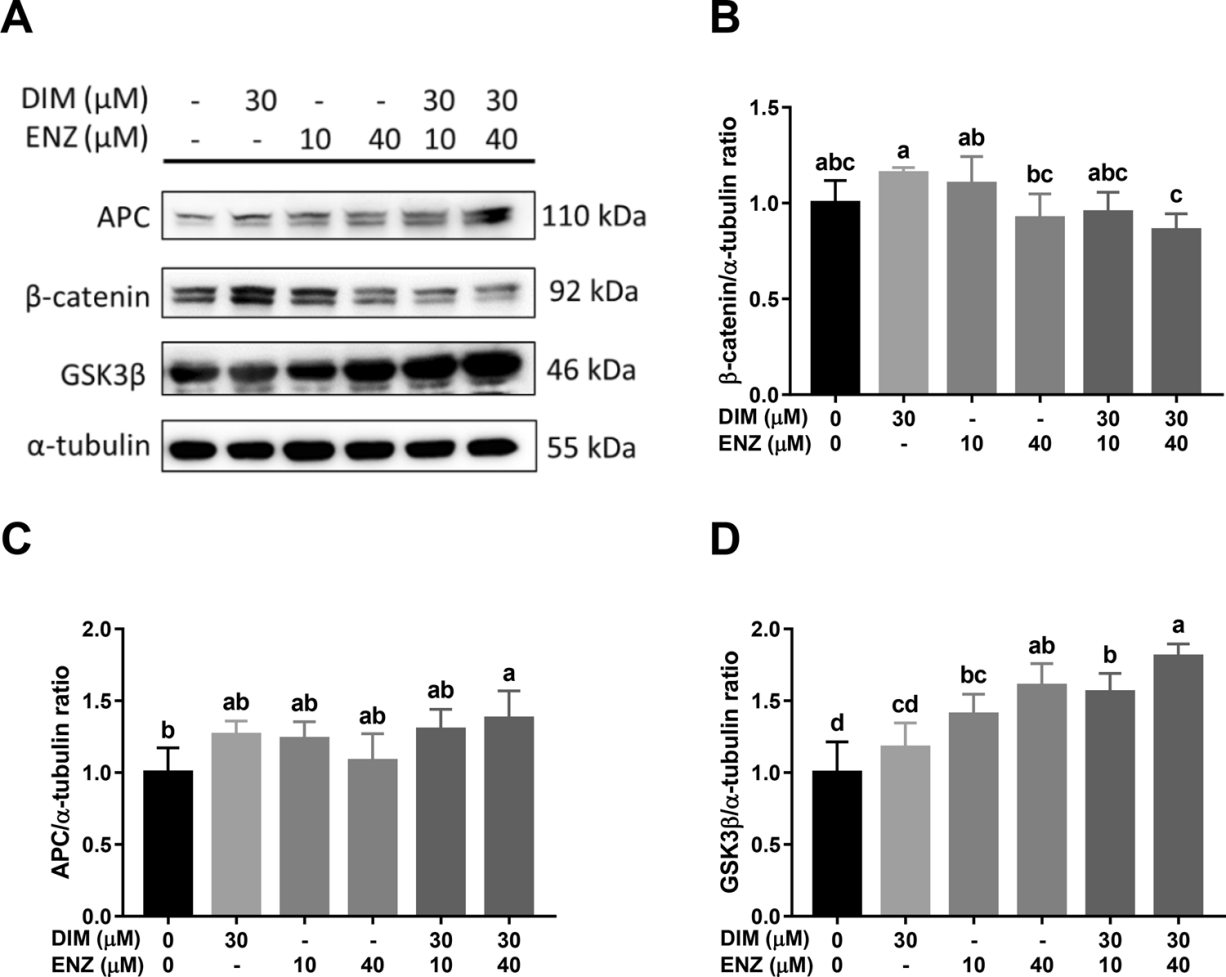
Inhibition of Wnt signaling by DIM and ENZ co-treatment in 22Rv1 cells at 72 h. Representative western blot image (**A**); quantitative results for β-catenin (**B**), APC (**C**), and GSK3β (**D**). *DIM* 3,3′-diindolylmethane; *ENZ* enzalutamide. ^a,b,c,d^Bars without the same letters at the top indicate statistically-significant differences between treatments when compared with each other (one-way ANOVA, Duncan’s new multiple range test, *p* < 0.05).

After 72 h, combined treatment with DIM and ENZ significantly increased the expressions of APC and GSK3β (Fig. 6A–C; *p* < 0.05 vs. control), but there was no significant difference in the expression of β-catenin (Fig. 6A,D). Compared with treatment of 22Rv1 cells with 10 μM ENZ alone, co-treatment with ENZ and DIM significantly upregulated the level of GSK3β and decreased the expression of β-catenin (Fig. 6; *p* < 0.05 vs. 10 μM ENZ). The results demonstrated that combined treatment with DIM and ENZ may inhibit Wnt signaling.

### DIM and ENZ downregulate AR and AR-v7 expressions

**Figure 7:**
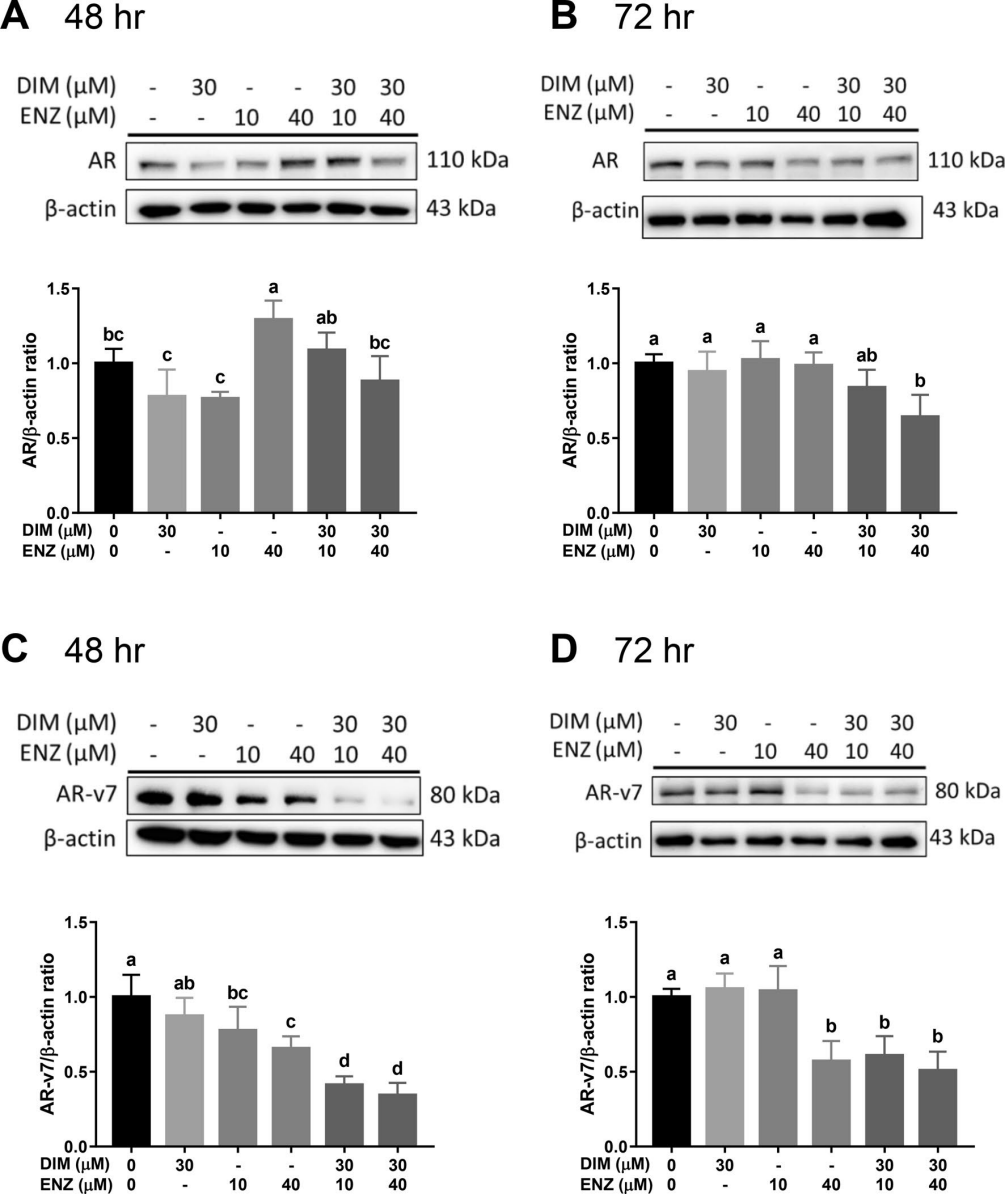
DIM and ENZ downregulate AR and AR-v7 protein expressions. AR expression at 48 (**A**) and 72 h (**B**); AR-v7 expression at 48 (**C**) and 72 h (**D**). *DIM* 3,3′-diindolylmethane; *ENZ* enzalutamide. ^a,b,c,d^Bars without the same letters at the top indicate statistically-significant differences between treatments when compared with each other (one-way ANOVA, Duncan’s new multiple range test, *p* < 0.05).

Aberrant AR signaling is considered a critical mechanism in CRPC. Moreover, a mechanism of cross-talk between Wnt and AR signaling has been identified. Therefore, to elucidate whether AR signaling is inhibited when Wnt signaling is inhibited, western blot analysis was employed to detect the expression of AR. After 48 h of treatment, 40 μM ENZ significantly increased the expression of AR (Fig. 7A; *p* < 0.05 vs. control); however, combined treatment with DIM and 40 μM ENZ significantly decreased the level of AR (Fig. 7A; *p* < 0.05 vs. 40 μM ENZ). After 72 h of treatment, exposure to DIM and 40 μM ENZ significantly downregulated the AR expression (Fig. 7B; *p* < 0.05 vs. control).

Besides AR overexpression, a high AR-v7 expression has been found in ENZ-resistant PCa, and AR-v7 may also activate AR signaling. Therefore, to examine the effect of DIM on AR-v7 expression, western blot analysis was used to detect the protein expression of AR-v7 in 22Rv1 cells. After 48 h of treatment, exposure to ENZ alone or in combination with DIM significantly decreased the AR-v7 expression (Fig. 7C; *p* < 0.05 vs. control). Compared with treatment of 22Rv1 cells with 10 μM ENZ alone, co-treatment with ENZ and DIM significantly downregulated the level of AR-v7 (Fig. 7C; *p* < 0.05 vs. 10 μM ENZ). After 72 h of treatment, 40 μM ENZ and combined treatment with DIM and ENZ significantly decreased the AR-v7 expression (Fig. 7D; *p* < 0.05 vs. control). The results indicated that co-treatment with DIM and ENZ may inhibit AR signaling by downregulating the expressions of AR and AR-v7.

### DIM and ENZ treatment alter EMT-related mRNA and protein expressions in PCa cells

**Figure 8:**
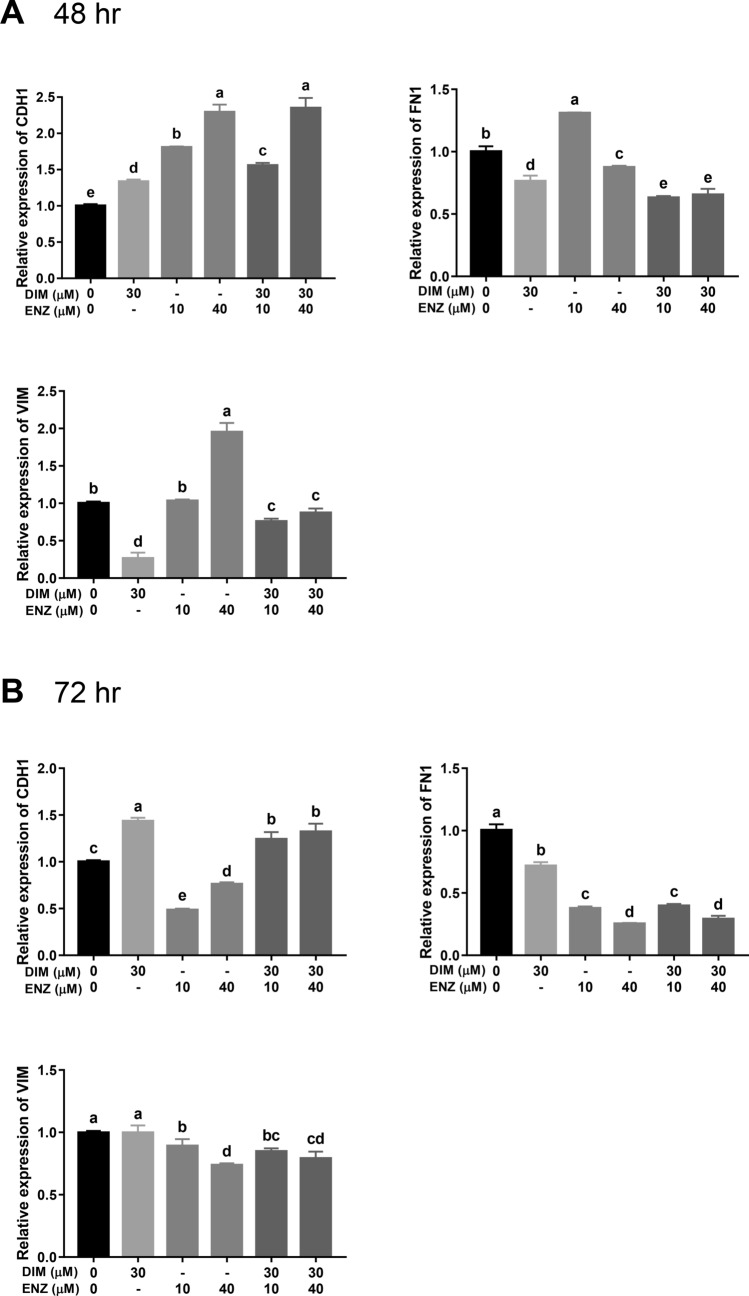
DIM and ENZ treatment alter EMT-related gene expressions in 22Rv1 cells at 48 (**A**) and 72 (**B**) h (n = 3). *DIM* 3,3′-diindolylmethane; *ENZ* enzalutamide; *VIM* Vimentin; *FN1* Fibronectin; *CDH1* E-cadherin. ^a,b,c,d^Bars without the same letters at the top indicate statistically-significant differences between treatments when compared with each other (one-way ANOVA, Duncan’s new multiple range test, *p* < 0.05).

The process of EMT plays a pivotal role in CRPC and is related to drug resistance. Moreover, Wnt signaling and AR signaling activate EMT in CRPC. To investigate the role of EMT in CRPC, qPCR was performed to examine the mRNA expressions of an epithelial marker (E-cadherin, CDH1) and mesenchymal markers (Fibronectin and Vimentin, FN1 and VIM). After treatment for 48 h, exposure to DIM or ENZ alone, and combined treatment with DIM and ENZ, significantly upregulated the expression of *CDH1* (Fig. 8A; *p* < 0.05 vs. control). On the other hand, cells treated with DIM alone and co-treated with DIM and ENZ exhibited significantly decreased expressions of *FN1* and *VIM*, but cells treated with 10 μM ENZ alone exhibited a significantly increased expression of *FN1* (Fig. 8A; *p* < 0.05 vs. control). Exposure to 40 μM ENZ significantly downregulated the level of *FN1* but increased the expression of *VIM* (Fig. 8A; *p* < 0.05 vs. control). Compared with treatment of 22Rv1 cells with 10 μM ENZ alone, co-treatment with ENZ and DIM significantly upregulated the level of *CDH1* and downregulated the levels of *FN1* and *VIM* (Fig. 8A; *p* < 0.05 vs. 10 μM ENZ).

After treatment for 72 h, exposure to DIM alone and combined treatment with DIM and ENZ significantly upregulated the expression of *CDH1*, but cells treated with ENZ alone exhibited a significantly decreased level of *CDH1* (Fig. 8B; *p* < 0.05 vs. control). Exposure to DIM or ENZ alone, and co-treatment with DIM and ENZ, significantly inhibited the *FN1* expression, but only ENZ treatment and combined treatment decreased the *VIM* expression (Fig. 8B; *p* < 0.05 vs. control). Compared with treatment of 22Rv1 cells with 10 μM ENZ alone, co-treatment with ENZ and DIM significantly upregulated the level of *CDH1* and downregulated the levels of *FN1* and *VIM* (Fig. 8B; *p* < 0.05 vs. 10 μM ENZ).

**Figure 9:**
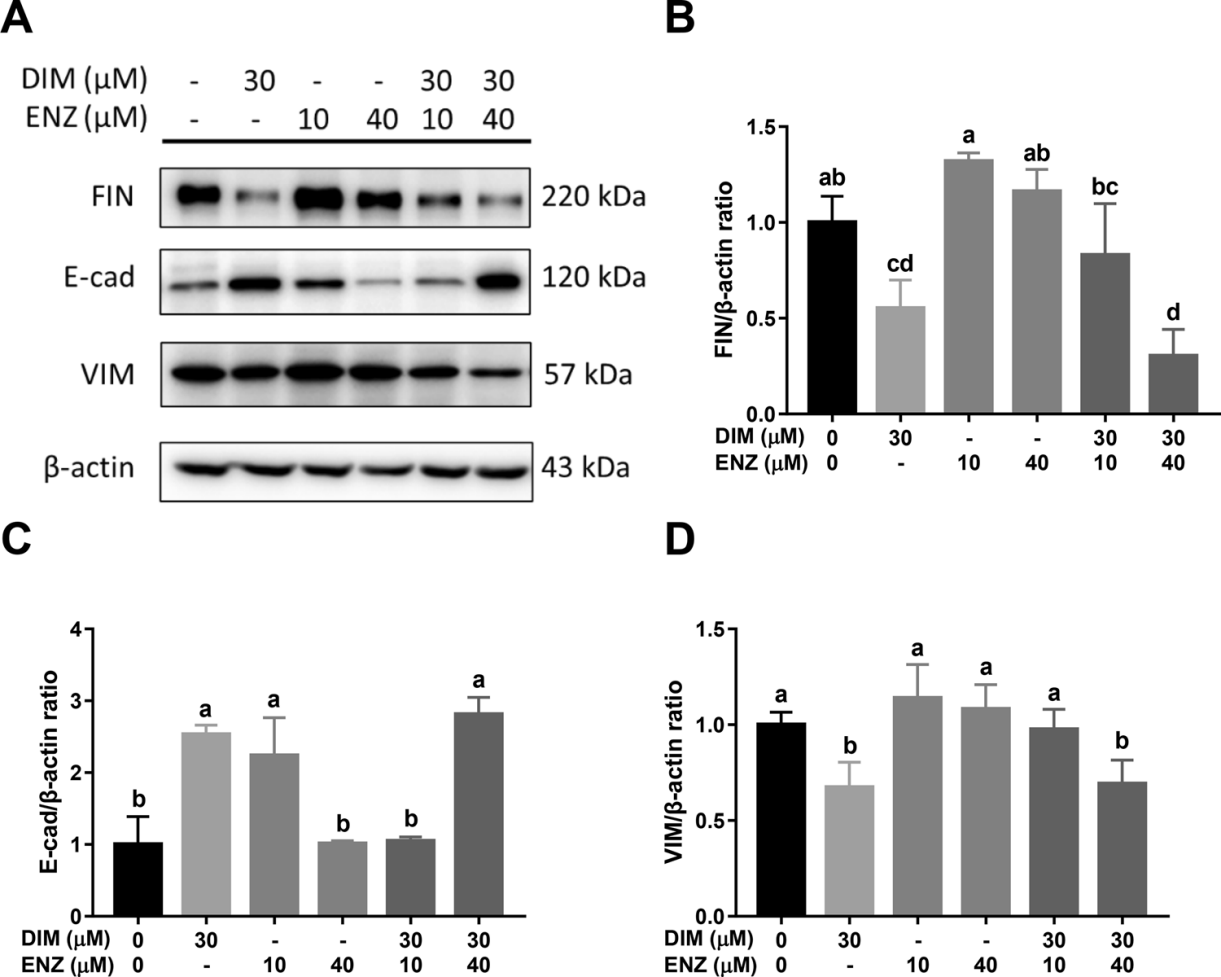
DIM and ENZ treatment alter EMT-related protein expressions in 22Rv1 cells at 48 h. Representative western blot image (**A**); quantitative results for FIN (**B**), E-cad (**C**), and VIM (**D**). *DIM* 3,3′-diindolylmethane; *ENZ* enzalutamide; *VIM* Vimentin; *FIN* Fibronectin; *E-cad* E-cadherin. Values are presented as means ± SD. ^a,b,c,d^Bars without the same letters at the top indicate statistically-significant differences between treatments when compared with each other (one-way ANOVA, Duncan’s new multiple range test, *p* < 0.05).

Furthermore, western blot analysis was employed to detect the protein expressions of E-cadherin (E-cad), Fibronectin (FIN) and Vimentin (VIM). After treatment for 48 h (Fig. 9A), exposure to DIM or 10 μM ENZ alone, and combined treatment with DIM and 40 μM ENZ, significantly upregulated the expression of E-cad (Fig. 9C; *p* < 0.05 vs. control). In contrast, cells treated with DIM alone and co-treated with DIM and 40 μM ENZ had significantly decreased expressions of FIN and VIM (Fig. 9B,D; *p* < 0.05 vs. control). Compared with treatment of 22Rv1 cells with 10 μM ENZ alone, co-treatment with DIM and ENZ significantly downregulated the expression of FIN (Fig. 9B; *p* < 0.05 vs. 10 μM ENZ).

**Figure 10:**
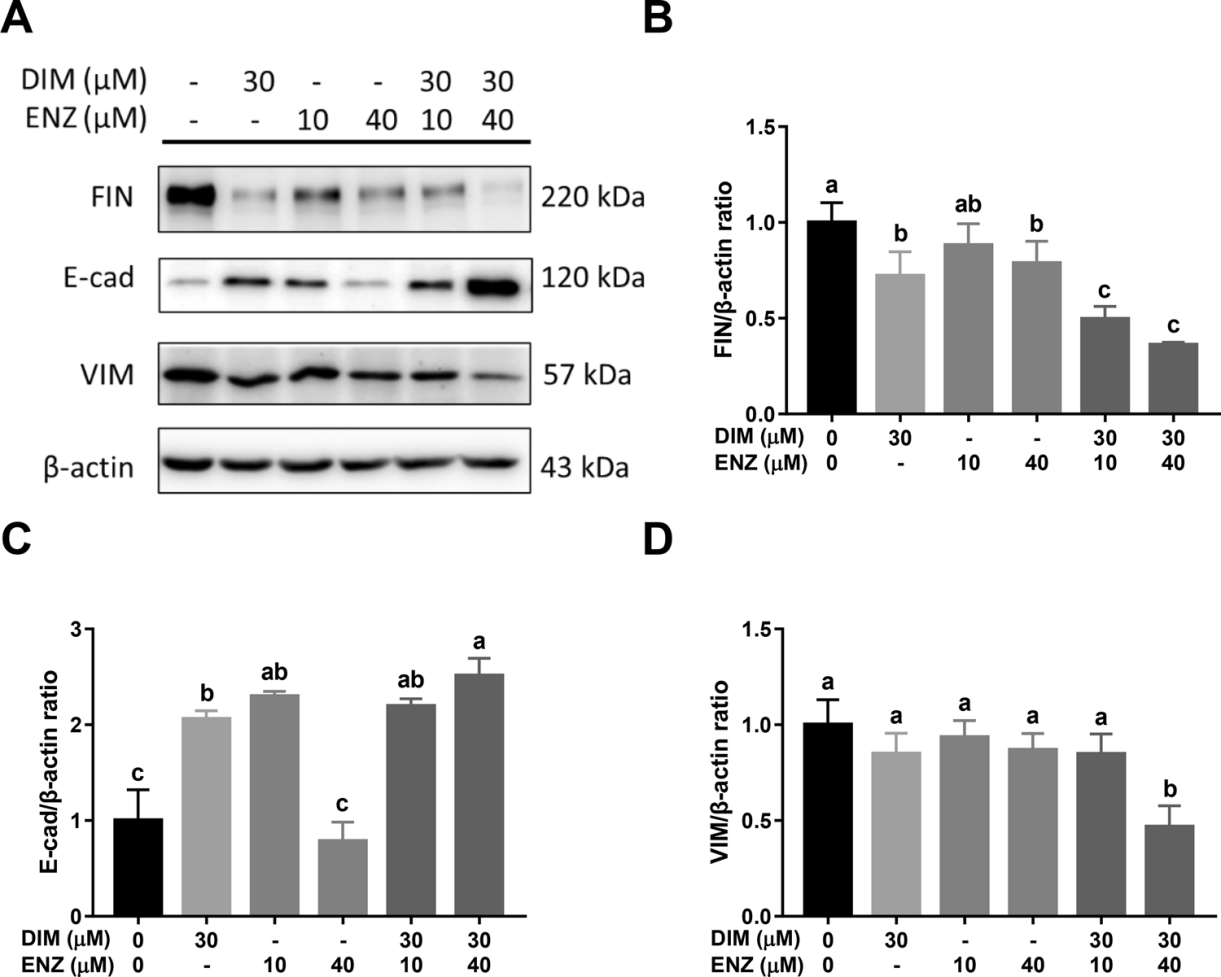
DIM and ENZ treatment alter EMT-related protein expressions in 22Rv1 cells at 72 h. Representative western blot image (**A**); quantitative results for FIN (**B**), E-cad (**C**), and VIM (**D**). *DIM* 3,3′-diindolylmethane; *ENZ* enzalutamide; *VIM* Vimentin; *FIN* Fibronectin; *E-cad* E-cadherin. Values are presented as means ± SD. ^a,b,c^Bars without the same letters at the top indicate statistically-significant differences between treatments when compared with each other (one-way ANOVA, Duncan’s new multiple range test, *p* < 0.05).

After treatment for 72 h (Fig. 10A), exposure to DIM and 10 μM ENZ alone and combined treatment with DIM and ENZ significantly increased the expression of E-cad (Fig. 10C; *p* < 0.05 vs. control). Exposure to DIM and 40 μM ENZ alone, and co-treatment with DIM and ENZ, significantly inhibited the FIN expression, but only combined treatment decreased the VIM expression (Fig. 10B,D; *p* < 0.05 vs. control). Compared with treatment of 22Rv1 cells with 10 μM ENZ alone, co-treatment with ENZ and DIM significantly decreased the expressions of FIN and VIM (Fig. 10B,D; *p* < 0.05 vs. 10 μM ENZ). The results revealed that co-treatment with DIM and ENZ possibly inhibits EMT and improves drug resistance.

### DIM and ENZ treatment do not promote cell cytotoxicity in PCa cells

**Figure 11:**
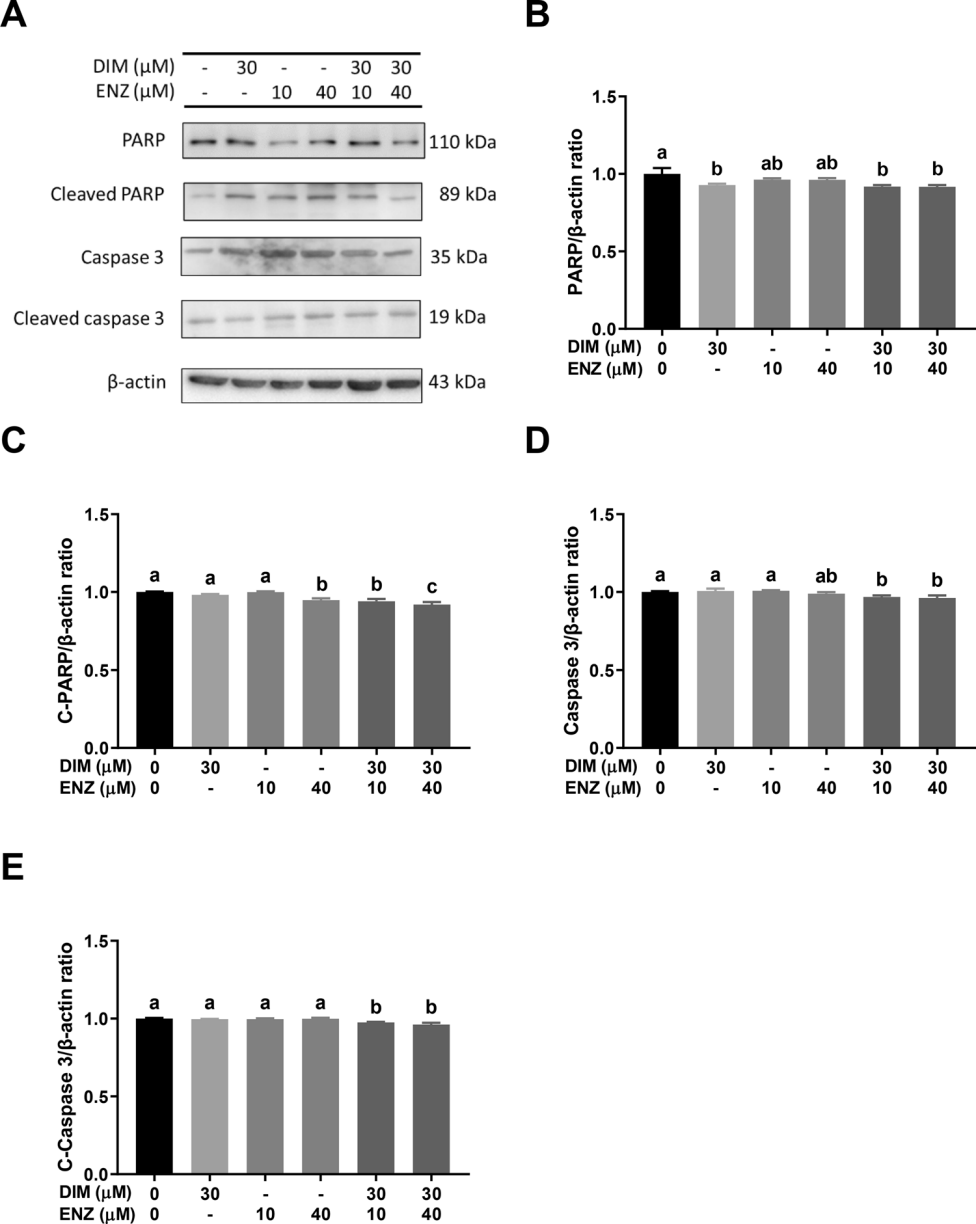
No evident cell cytotoxicity in 22Rv1 cells treated with DIM and ENZ for 48 h. Representative western blot image (**A**); quantitative results for PARP (**B**), C-PARP (**C**), Caspase 3 (**D**) and C-Caspase 3 (**E**). *DIM* 3,3′-diindolylmethane; *ENZ* enzalutamide; *PARP* poly (ADP-ribose) polymerase; *C-PARP* cleaved poly (ADP-ribose) polymerase; *C-Caspase 3* cleaved caspase 3. Values are presented as means ± SD. ^a,b,c^Bars without the same letters at the top indicate statistically-significant differences between treatments when compared with each other (one-way ANOVA, Duncan’s new multiple range test, *p* < 0.05).

**Figure 12:**
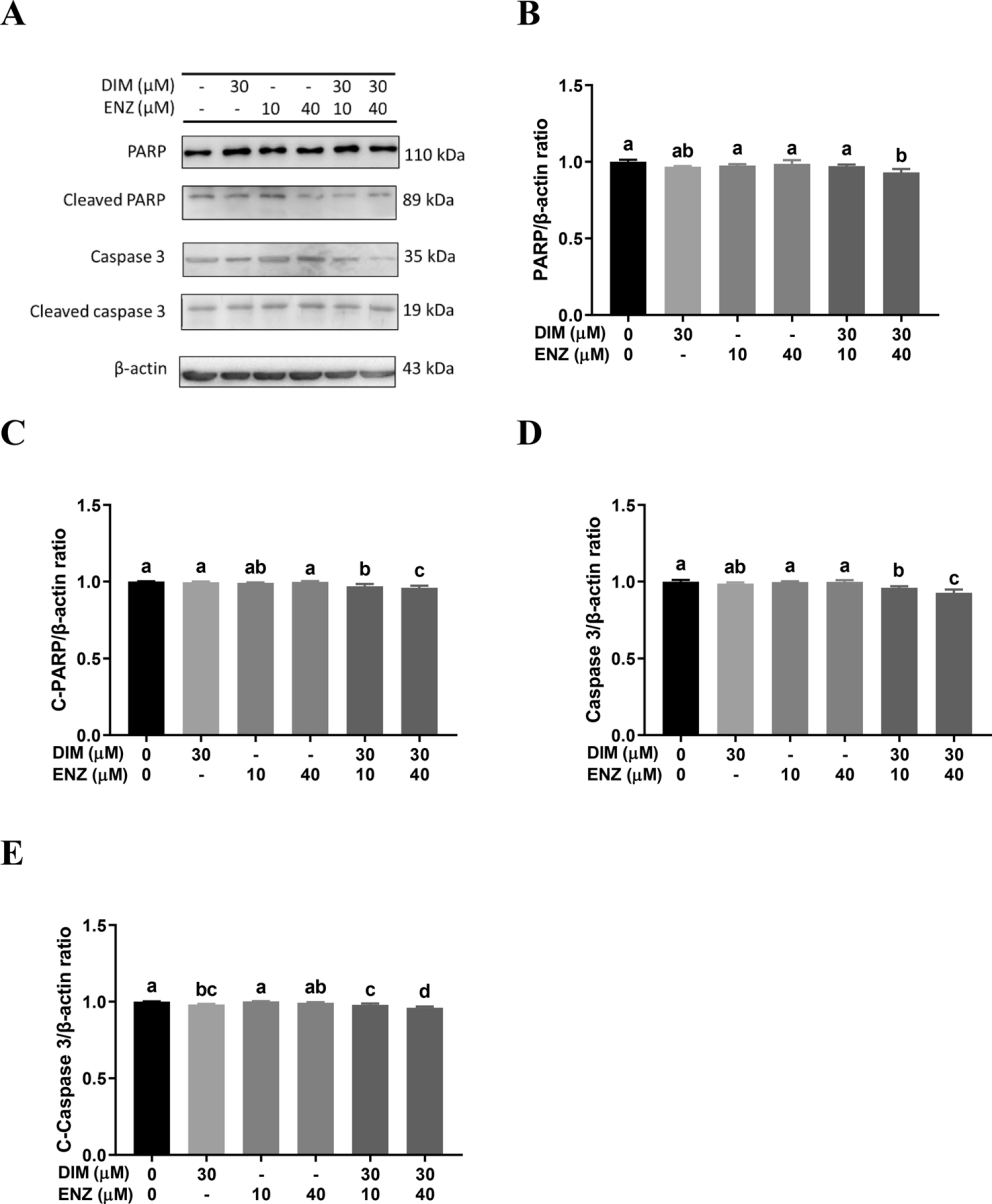
No evident cell cytotoxicity in 22Rv1 cells treated with DIM and ENZ for 72 h. Representative western blot image (**A**); quantitative results for PARP (**B**), C-PARP (**C**), Caspase 3 (**D**) and C-Caspase 3 (**E**). *DIM* 3,3′-diindolylmethane; *ENZ* enzalutamide; *PARP* poly (ADP-ribose) polymerase; *C-PARP* cleaved poly (ADP-ribose) polymerase; *C-Caspase 3* cleaved caspase 3. Values are presented as means ± SD. ^a,b,c,d^Bars without the same letters at the top indicate statistically-significant differences between treatments when compared with each other (one-way ANOVA, Duncan’s new multiple range test, *p* < 0.05).

To ascertain whether combined treatment with DIM and ENZ had a cytotoxic effect on PCa cells, 22Rv1 cells were treated with DIM and ENZ alone and together for 48 and 72 h. The results showed that treatment with DIM for 48 and 72 h significantly inhibited PARP and c-Caspase 3, respectively, and treatment of 22Rv1 cells with DIM and ENZ together for 48 and 72 h significantly decreased the levels of PARP, c-PARP, Caspase 3 and c-Caspase 3 (Figs. 11 and 12; *p* < 0.05 vs. control). The results showed that ENZ and DIM treatment may not enhance cell cytotoxicity.

**Figure 13:**
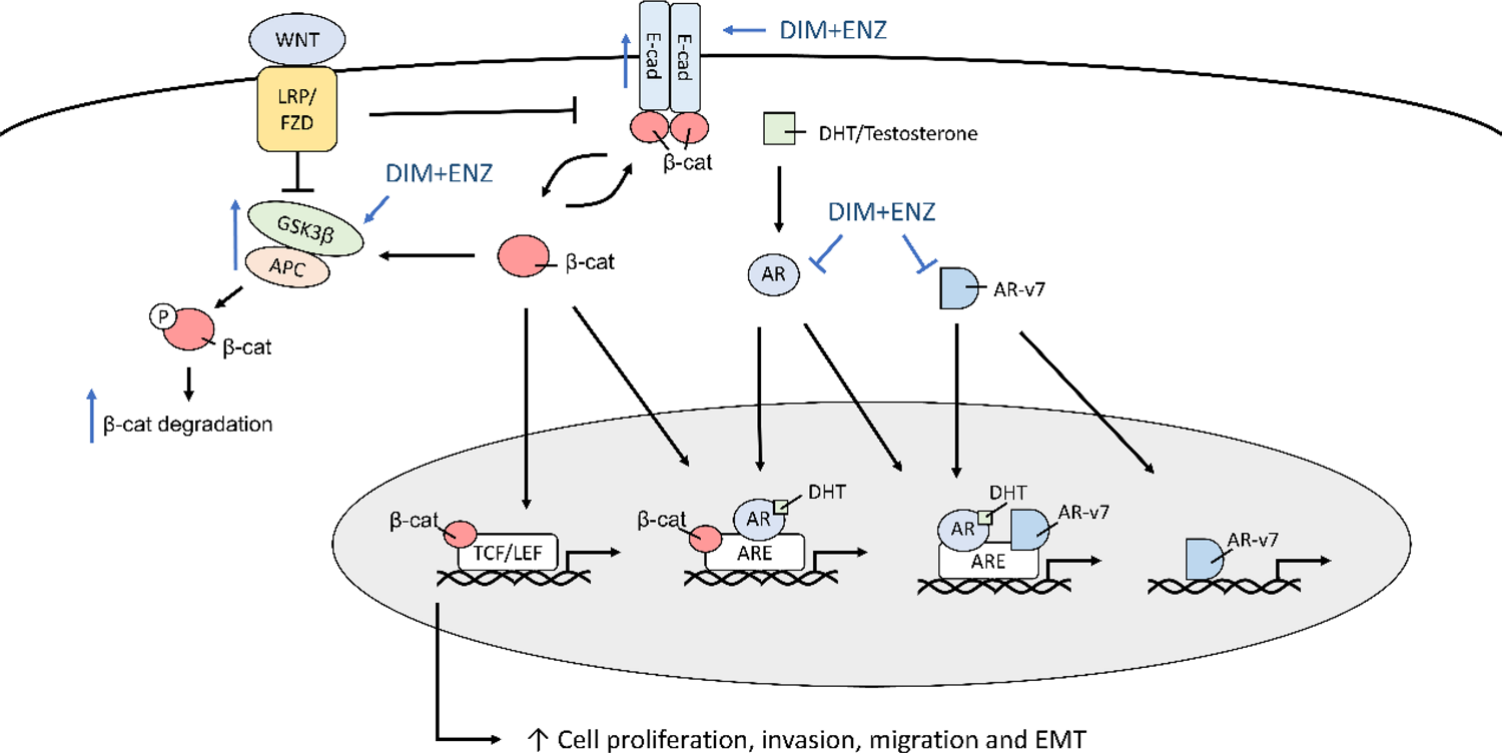
Co-treatment with DIM and ENZ regulates Wnt and AR signaling. *AR* androgen receptor, *ARE* androgen receptor element, *DHT* dihydrotestosterone, *APC* adenomatous polyposis coli protein, *GSK3* glycogen synthase kinase‑3, *TCF/LEF* T-cell factor/lymphoid enhancer factor transcription factors.

According to these results, we attempted to outline a possible model to elucidate the role of DIM in an ENZ-resistant prostate cancer cell line (Fig. 13). The cell treatment with 10 μM ENZ is as a model of ENZ resistant control group. Our results showed that co-treatment with DIM and ENZ resulted in the strongest inhibition of cell proliferation, colony formation, invasion and migration. Furthermore, co-treatment with DIM and ENZ significantly increased the AR and AR-v7 protein expressions to inhibit AR signaling (vs. control and 10 μM ENZ). In addition, treatment with DIM and ENZ for 72 h significantly increased the expressions of APC and GSK3β, and decreased the expression of β-catenin (vs. control), which indicated that co-treatment suppressed Wnt signaling. Both signaling pathways are related to the progression of EMT, and the results showed that co-treatment significantly increased the expression of E-cadherin and decreased the expressions of Fibronectin and Vimentin to suppress EMT (vs. control and 10 μM ENZ).

## Discussion

The 22Rv1 cell line is a CRPC cell line that expresses AR and has the highest expression of AR-v7 as compared with other CRPC cell lines^37,38^. In comparison with these other cell lines, 22Rv1 cells have higher levels of expression of AR and AR-v7, the other cells barely expressing AR-v7 at all. In the present study, we aimed to investigate whether inhibition of AR and AR-v7 expressions by DIM is a main cause of enzalutamide resistance. Therefore, we employed 22Rv1 cells as the main model in this study.

The results of previous studies and the present study showed that treatment of 22Rv1 cells with ENZ (0–40 μM) did not significantly inhibit cell proliferation, proving that 22Rv1 is ENZ-resistant^39,40^. Therefore, in this study, we used 22Rv1 cells to ascertain whether DIM treatment could improve ENZ resistance. According to previous studies, DIM suppresses cell proliferation in various cancers, including prostate cancer, breast cancer and ovarian cancer^41,42^. Exposure of LNCaP, DU145, PC-3, C4-2B and 22Rv1 PCa cell lines to DIM or BR-DIM was found to reduce the cell viability^43–46^. Moreover, BR-DIM inhibited PCa cell invasion and proliferation by decreasing platelet-derived growth factor expression and activity^47^. In addition, Admad et al.^48^ showed that treatment of PC-3 cells with 25 μM BR-DIM downregulated uPA by decreasing the expressions of VEGF and MMP-9 to inhibit cell proliferation, invasion and migration. The results of this study showed that DIM had significant anticancer effects on 22Rv1 cells, and combined treatment with DIM and ENZ had stronger effects in terms of inhibiting cancer cell proliferation, colony formation, invasion and migration. DIM re-sensitizes ENZ-resistant cells to ENZ treatment, proving that it may be able to be developed as an adjuvant therapy for PCa.

In the clinical treatment of PCa, repressing AR signaling is a critical strategy^6,7^. ENZ is one of the newer and potentially more effective AR-targeting agents^49^; however, the response to ENZ is not permanent, and almost all patients eventually develop resistance to ENZ, demonstrating that targeting AR signaling alone is not sufficient for CRPC therapy^6,10^. Lee et al.^50^ revealed that Wnt signaling might be active in PCa cells after ADT, partly due to enforcement of interaction between β-catenin and TCF. In androgen-independent PCa cell lines, the combination of a GSK3β inhibitor or APC knockdown with ENZ increases growth inhibition through repressing both AR signaling and Wnt signaling^50^. In addition, overexpressions of *AR* and *CTNNB1* (β-catenin) were found in ENZ-resistant PCa cells, and the combination of β-catenin inhibitor ICG001 with ENZ improved ENZ resistance^22^. A previous study indicated that exposure of LNCaP and C4-2B PCa cells to 50 μM BR-DIM decreased the phosphorylation of GSK3β, and then inhibited nucleus translocation of β-catenin; in addition, it caused FOXO3a to bind to the p27^kip1^ promoter rather than the AR promoter^51^. Leem et al.^52^ showed that treatment of colon cancer cells with DIM downregulated the expressions of *β-catenin*, *Myc* and *FOS*, which are related to cancer prevention and prognosis. In gastric cancer cells, treatment with different concentrations of DIM produces contrary results. A high level of DIM (30 μM) resulted in inhibition of cell proliferation, but a low level (1 and 10 μM) activated Wnt4 signaling, which enhanced the progression of gastric cancer^53^. In the present study, we decided to use a high level of DIM to avoid any adverse influence, and our results showed that DIM did not significantly inhibit Wnt signaling, but combined treatment with DIM and ENZ significantly inhibited Wnt signaling through upregulating the expressions of APC and GSK3β and downregulating the β-catenin expression.

In addition, there is a connection between AR signaling and Wnt signaling. In normal prostate epithelial cells, androgen modulates the activation of AR signaling^19,28^. However, in PCa, the increased level of β-catenin leads to the formation of β-catenin–AR complexes, and the formation of β-catenin–TCF complexes is preferred under the condition of absence of androgen, which express target genes, including *AR*, *c-myc*, *MMP7*, and *VEGF*^19,28^. DIM has been proven to be a strong androgen antagonist that is a competitive inhibitor of DHT binding to AR^54^. DIM binds to AR directly, and then inhibits nucleus translocation of AR and transcription activation^54^. However, Palomera-Sanchez et al.^55^ treated LNCaP cells with DIM for 48 h, and the results showed that DIM inhibited the protein expression of AR, but increased the mRNA expression of *AR*. Although the mRNA expression of *AR* increased, chromatin modifications at its promoter region had no significant effects^55^. In our study, DIM downregulated the protein expression of AR, but upregulated its mRNA expression (data not shown), consistent with previous studies. DIM treatment might cause AR turnover by inducing AR-protein instability. Several studies have revealed that AR overexpression and increased expressions of AR-Vs alleviate the efficacy of ENZ and induce progression of PCa^11,23,27,56,57^. Of the variants, AR-v7 is the most common in patients with ENZ resistance, and activates ligand-independent AR signaling^58–60^. Ong et al.^61^ showed that exposure of PCa cells to BR-DIM for 96 h significantly downregulated the AR-v7 mRNA expression. However, DIM did not significantly decrease the level of AR-v7 protein expression in this study. Although the effects of DIM on AR-v7 expression were not significant, combined DIM and ENZ treatment significantly inhibited the AR-v7 expression.

Recent studies have indicated that EMT is involved in PCa progression, migration and therapy resistance, but the effects of ADT on EMT are still unclear^62^. There are several mechanisms, including AR signaling and Wnt signaling, that activate the process of EMT^62^. Numerous molecular processes mediate the process of EMT, which is associated with loss of epithelial factors (e.g., E-cadherin and zona occludens protein-1) and gain of mesenchymal factors (e.g., N-cadherin, Fibronectin and Vimentin)^24^. In normal prostate tissue, canonical AR signaling suppresses EMT^24^; however, a previous study showed that androgen deprivation increased the expressions of AR and AR-Vs, and upregulated EMT markers (ZEB1, Snail, Twist, N-cadherin and Vimentin), which caused therapy resistance^63^. Li et al.^63^ reported that BR-DIM increased the *E-cadherin* expression and decreased the *vimentin* expression by inhibiting miR-92a expression, and then suppressed the process of EMT. In addition, miR-34a, miR-27b, miR-320m, miR-200 and let-7 were re-expressed by BR-DIM, which then repressed the expressions of AR, AR-Vs and EMT marker^45,61,64^. In the present study, DIM increased the E-cadherin expression and decreased the expressions of Fibronectin and Vimentin, then inhibited the process of EMT, which was consistent with the results of previous studies. Compared with treatment with ENZ alone, combined treatment with DIM and EMT resulted in stronger inhibition of EMT, which confirmed that DIM may mediate ENZ resistance by inhibiting EMT.

Although our results demonstrated that DIM could inhibit both Wnt signaling and AR signaling, the inhibition effects were not as obvious as those reported in previous studies, which might be because most of those studies used BR-DIM, rather than DIM. DIM is stable under acidic conditions, but the administration of DIM to female Sprague–Dawley rats by the i.p. route has better effects than the oral route^65^. Therefore, in order to enhance bioavailability, a formulation of DIM (BR-DIM) was developed to contain small particles of DIM within a water-soluble matrix, containing α-tocopherol polyethylene glycol succinate and phosphatidyl choline, by solubility-enhancing microencapsulation technology^66^. Anderton et al. administered DIM and BR-DIM to mice and then analyzed the concentration of DIM in the plasma and liver, kidney and lung tissues. The results showed that BR-DIM exhibited an approximate 50% higher bioavailability than DIM^66^. Moreover, in clinical studies, adjuvant intervention in the early stage of PCa has been performed using BR-DIM, and was proven to be an effective method by which to improve the therapeutic outcome^34^.

In our study, DIM was effective in ameliorating carcinogenesis through modulating AR signaling, Wnt signaling and EMT in ENZ-resistant cells. The findings may have several implications for the use of DIM as a dietary supplement or a therapeutic agent. The results only relate to patients who have already exhibited ENZ resistance, and in future study, cells could be treated with ENZ for a long duration in order to build a model of long-term ENZ resistance to solve the clinical problem. Furthermore, transcriptional activation of ARE and TCF directly influences AR and Wnt signaling, and can therefore be analyzed to confirm the results in future study.

## Conclusion

Combined treatment with DIM and ENZ significantly inhibited the proliferation, invasion and migration of 22Rv1 cells. DIM could regulate Wnt signaling to inhibit the recurrence of AR signaling by reducing the expressions of AR and AR-v7. In addition, inhibition of these two signaling pathways is related to suppression of the EMT process. Our results demonstrated that treatment with DIM, or a combination of DIM and ENZ, could potentially represent other ADTs.

## Methods

### Cell line and reagents

22Rv1 (ATCC CRL-2505) is an androgen-independent PCa cell line that expresses AR and AR-v7, and is often used as a CRPC model. Cells were cultured in RPMI 1640 (Life Technologies/Gibco, Waltham, MA, USA) supplemented with 10% fetal bovine serum (FBS) (Gibco, Waltham, MA, USA), sodium pyruvate and 0.2% penicillin/streptomycin (Biological Industries, Cromwell, CT, USA) in a humidified incubator containing 5% CO_2_ at 37 °C. DIM (Sigma-Aldrich, St. Louis, MO, USA) and ENZ (Toronto Research Chemicals, North York, ON, Canada) were dissolved in dimethyl sulfoxide (DMSO) and diluted in medium before use.

### Cell proliferation assay (MTS assay)

Cells were seeded at 5 × 10^4^ cells per well in 96-well plates and grown for 24 h, following which different concentrations of DIM and ENZ were added and incubated for 48 and 72 h. Viable concentrations were tested using a CellTiter 96 Aqueous One Solution Cell Proliferation Assay (Promega, Durham, NC, USA). Cell viability was analyzed from absorbance readings at 490 nm.

### Colony formation

The anchorage independent growth (AIG) assay of colony formation is performed in soft agar, and detects malignant transformation of cells. 7 × 10^3^ cells were mixed with 0.7% melting agar (Invitrogen, Carlsbad, CA, USA), and the mixtures were then placed on a solidified layer of 0.5% agar made from 1% melting agar, 2X RPMI and 20% FBS in a 6-well plate. The plate was incubated for 24 h, and 2 ml RPMI with different concentrations of DIM and ENZ were added. Fresh medium was added every 7 days during the different treatments. After incubation for 28 days, colonies were stained with crystal violet for 1 h, then captured and counted using Cell^3^iMager Neo (SCREEN Holdings Co., Rolling Meadows, IL, USA).

### Wound-healing

A wound-healing assay was employed to evaluate cell migration using a culture insert (ibidi GmbH, Munich, Germany). First, the culture insert was placed on a 24-well plate, and 10^6^ cells were seeded onto the insert. After the cells had grown in the medium until approximate confluence, the culture insert was removed and washed with PBS. Under varying conditions, different treatments were added to the well, and microscopy was employed to capture images at different time points. Images were analyzed using Image J (National Institutes of Health, Bethesda, MD, USA).

### Cell migration and invasion assays

Cell migration and invasion assays were performed using Transwell chambers with an 8.0-µm-pore PET membrane (Becton Dickinson Biosciences, San Jose, CA, USA USA). Transwell chambers were coated with 15 μL Matrigel (Becton Dickinson Labware, Bedford, MA, USA) for the invasion assay, while uncoated chambers were used for the migration assay. Cells at densities of 2 × 10^5^ and 10^5^ for the invasion and migration assays, respectively, were resuspended in 300 μL serum-free medium with different treatments and then added to the upper chamber. Then, 800 μL medium containing 10% FBS were added to the lower chamber. After 24 h of incubation, cells were fixed with 1% paraformaldehyde at 4 °C for 24 h and then stained with Giemsa (Merck, Darmstadt, Germany) for 2 min. Images were captured using a microscope, and invaded cells were counted using Image J (National Institutes of Health)^16,67,68^.

### Quantitative polymerase chain reaction (qPCR)

Cells were treated with different concentrations of DIM and ENZ for 48 and 72 h, and then total RNA was extracted using a RNeasy Plus Mini Kit (QIAGEN, Hilden, Germany). cDNA was generated from 2 μg of total RNA using SuperScript III Reverse Transcriptase (Invitrogen, Karlsruhe, Germany). qPCR analysis was performed using 2X SYBR Green I master on a LightCycler 480 system (Roche, Penzberg, Germany). The data were analyzed using the 2^−ΔΔCt^ method. The gene expression levels were normalized to GAPDH, and the results are presented as the relative fold change compared with the control. The sequences of the primers used are presented in Supplementary Table 1^16,67^.

### Western blot analysis

After treatment with different concentrations of DIM and ENZ for 48 and 72 h, cells were washed with PBS three times and then lysed in RIPA buffer (Thermo Scientific, Waltham, MA, USA), protease inhibitor and phosphatase inhibitor. The lysates were cleared by centrifugation at 4 °C for 30 min at 14,000 rpm, and the concentrations of proteins were measured using a BCA protein assay kit (PIERCE Biotechnology, Rockford, IL, USA). Equal amounts of the proteins were loaded per well, then separated using 8% SDS-PAGE gels and transferred onto polyvinyl difluoride (PVDF) membranes (Millipore, Burlington, MA, USA). The PVDF membranes were blocked with 5% non-fat milk at room temperature for 1 h and then incubated with primary antibodies (Supplementary Table 2) overnight at 4 °C. Following three washes with 0.1% tris-buffered saline with Tween 20 (TBST) buffer (BioMan, Taiwan), the membranes were incubated with the appropriate horseradish peroxidase (HRP)-conjugated secondary antibody for 1 h at room temperature, and detection was performed using enhanced chemiluminescence (ECL, Bio-Rad, Hercules, CA, USA). Images were captured with SPOT Xplorer and quantitated using Image J (National Institutes of Health)^16,67,69^.

### Statistical analysis

The results are presented as the mean ± SD of at least three experiments. The data were analyzed using one-way analysis of variance (ANOVA) with Duncan’s multiple range test to determine significant differences between groups. These analyses were performed using SAS 9.4 software (SAS Institute Inc., Cary, NC, USA). *p* < 0.05 was considered to indicate a statistically-significant difference^16,67^.

## Supplementary Information

Supplementary Figures.

Supplementary Tables.
