# Supplementary material for: Regulation of carcinogenesis and mediation through Wnt/β-catenin signaling by 3,3′-diindolylmethane in an enzalutamide-resistant prostate cancer cell line
Source: Sci Rep. 2021 Jan 13;11:1239. doi: 10.1038/s41598-020-80519-3 (PMC7806813; doi:10.1038/s41598-020-80519-3)
Supplement: Supplementary file 1 — Supplementary Figures. [file 41598_2020_80519_MOESM1_ESM.docx]

**Regulation of carcinogenesis and mediation through Wnt/β-catenin signaling by 3,3’-diindolylmethane in an** **enzalutamide- resistant prostate cancer cell line**

**Running title:** DIM in ENZ-resistant prostate cancer

Chih-Wei Tsao^1^_,_ Jia-Sin Li^2^, Ya-Wen Lin^3^, Sheng-Tang Wu^1^, Tai-Lung Cha^1^, Chin-Yu Liu^2^*

^1^ Division of Urology, Department of Surgery, Tri-Service General Hospital, National Defense Medical Center, Taipei, Taiwan

^2^ Department of Nutritional Science, Fu Jen Catholic University, Taipei, Taiwan

^3^ Department of Microbiology and Immunology, National Defense Medical Centre, Taipei, Taiwan

*Corresponding author: Chin-Yu Liu, Ph.D.

(A) APC_48 hr


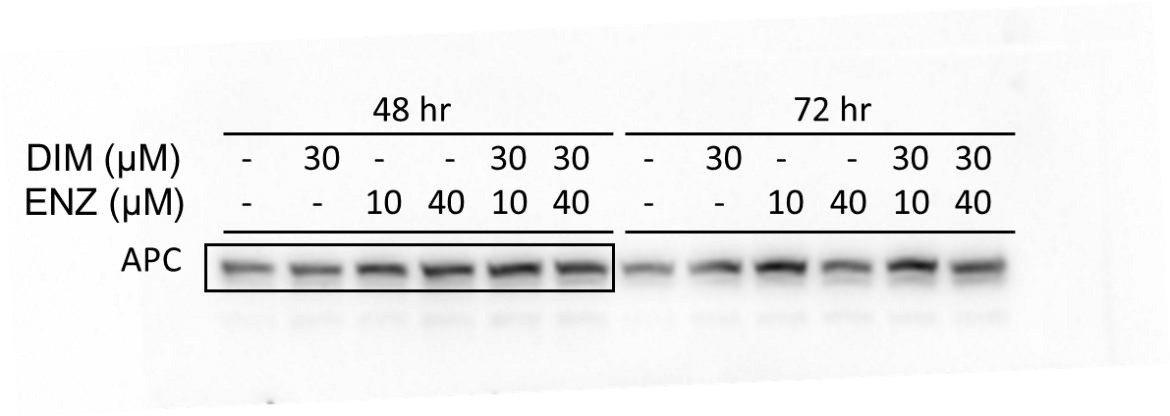


(B) β-catenin_48 hr


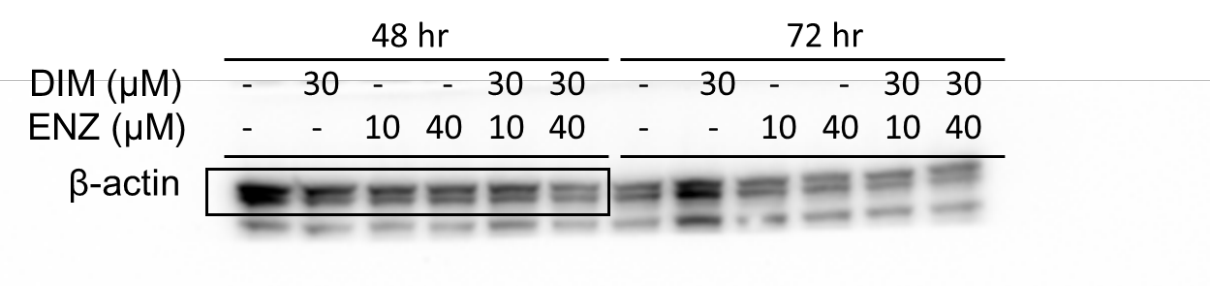


(C) GSK3β_48 hr


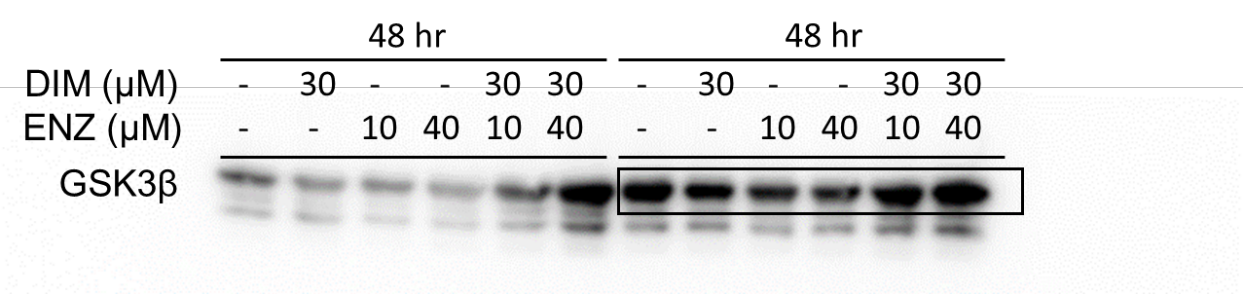


(D) α-tubulin_48 hr


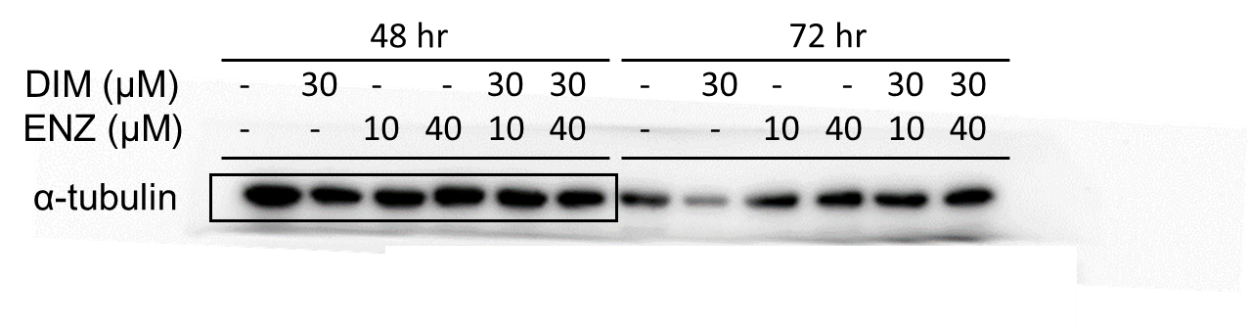


Supplementary Figure 1. Full-length western blot gels of Figure 5. Cropped gels are marked with a black box. APC (A), β-catenin (B), GSK3β (C), and α-tubulin (D). Image resolution = 600 dpi.

(A) APC_72 hr


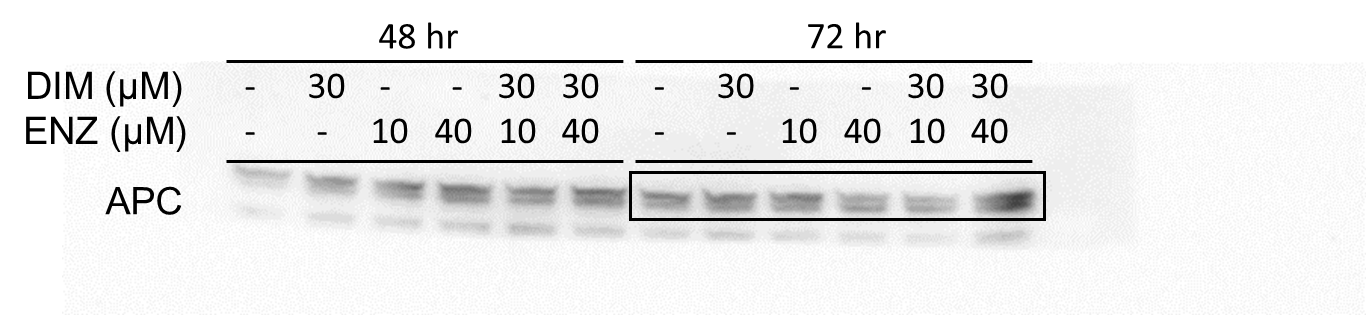


(B) β-catenin_72 hr


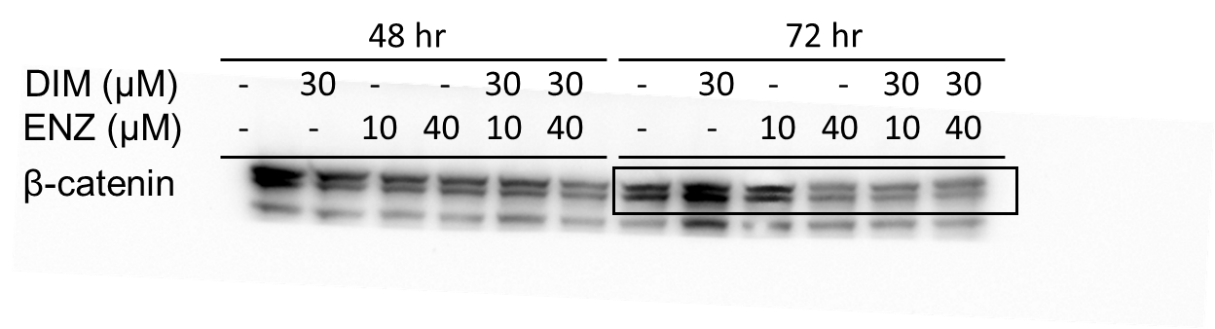


(C) GSK3β_72 hr


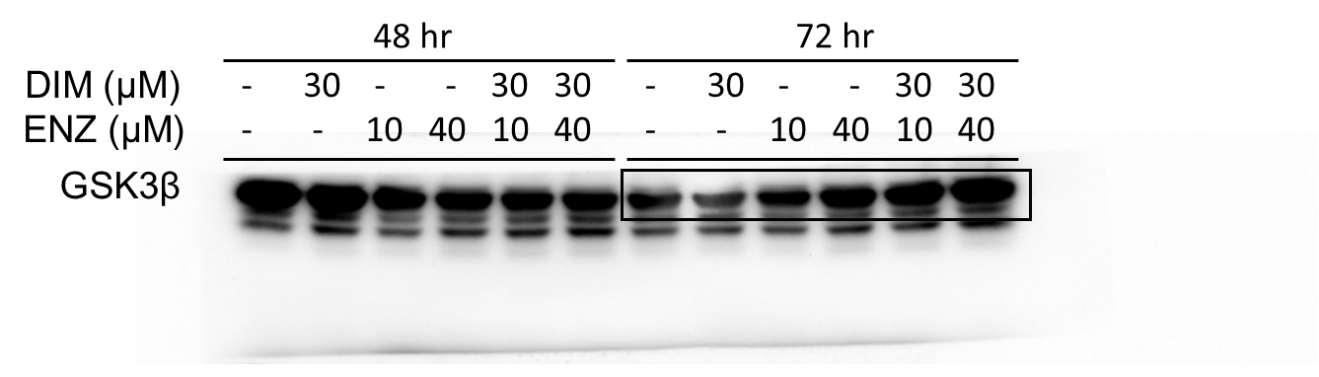


(D) α-tubulin_72 hr


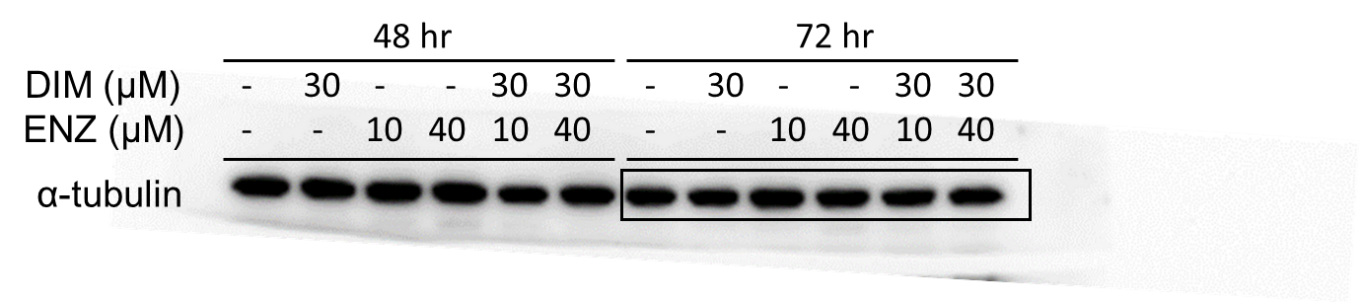


Supplementary Figure 2. Full-length western blot gels of Figure 6. Cropped gels are marked with a black box. APC (A), β-catenin (B), GSK3β (C), and α-tubulin (D). Image resolution = 600 dpi.

(A) AR_48 hr


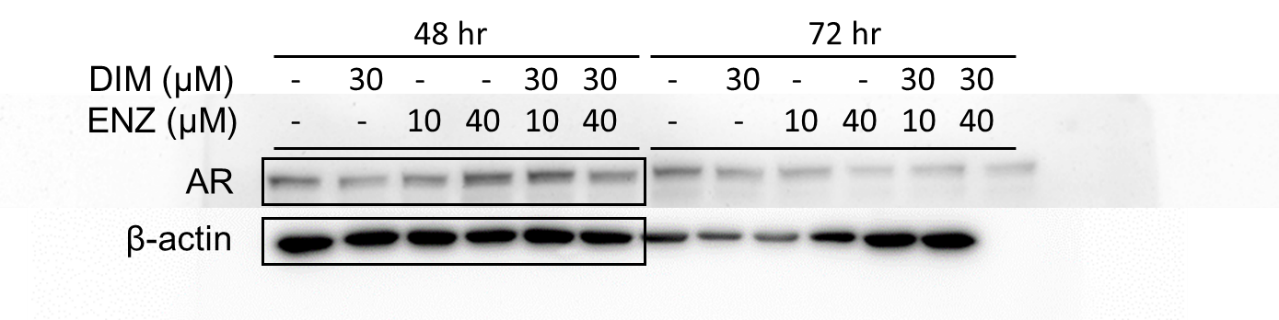


(B) AR_72 hr


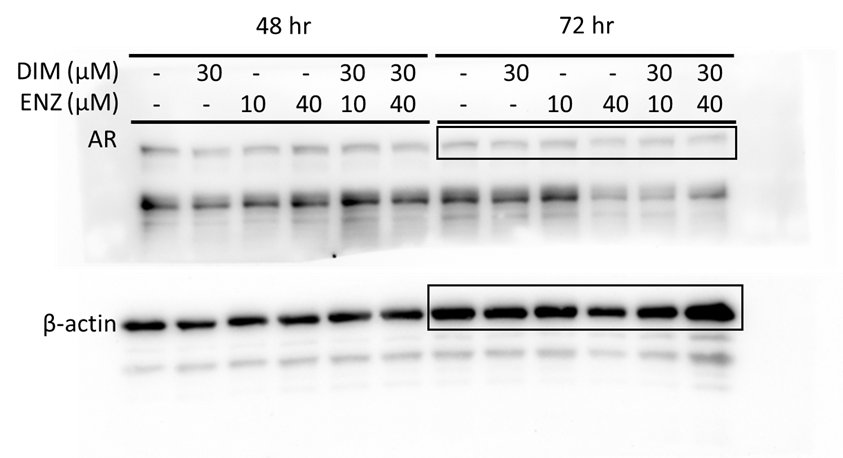


(C) AR-v7_48 hr


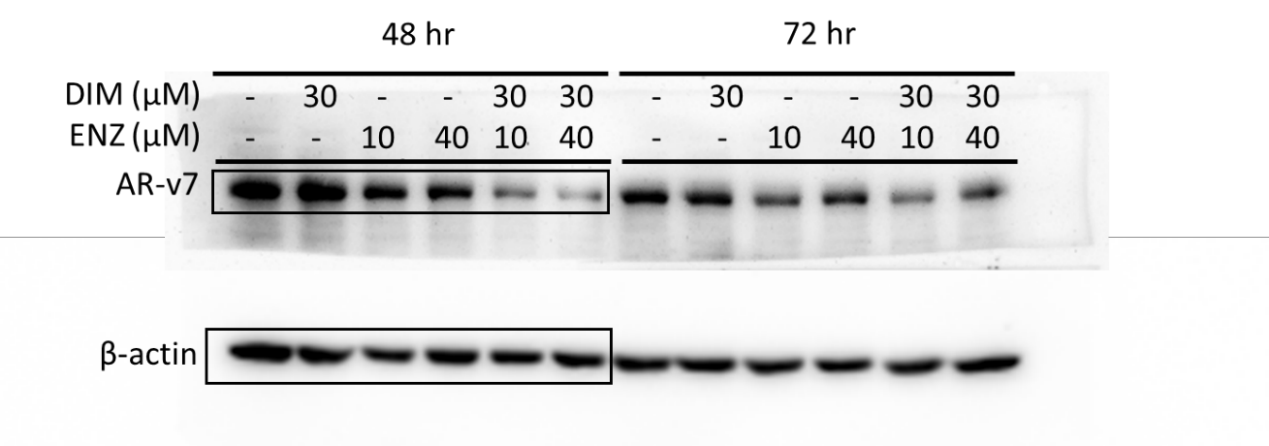


(D) AR-v7_72 hr


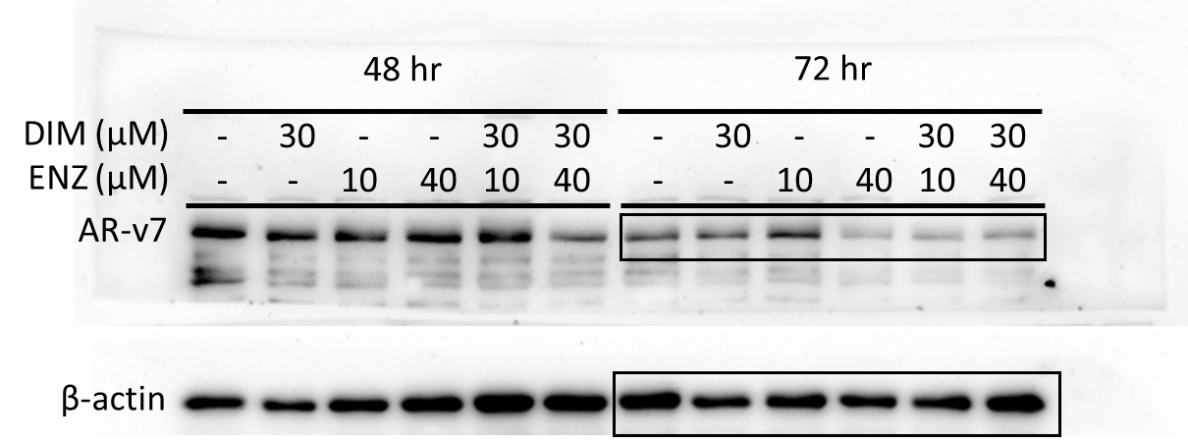
Supplementary Figure 3. Full-length western blot gels of Figure 7. Cropped gels are marked with a black box. AR (A) and (B), AR-v7 (C) and (D). Image resolution = 600 dpi.

(A) FIN_48 hr


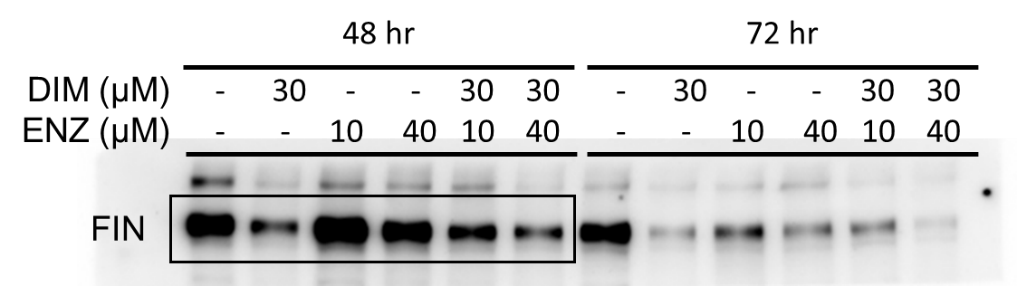


(B) E-cadherin_48 hr


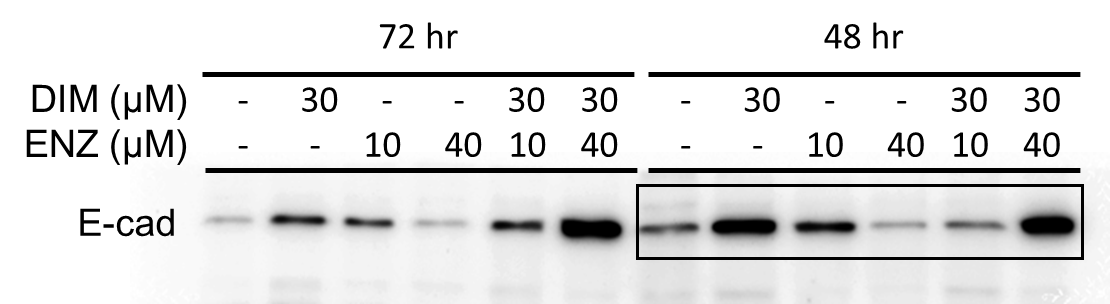


(C) VIM_48 hr


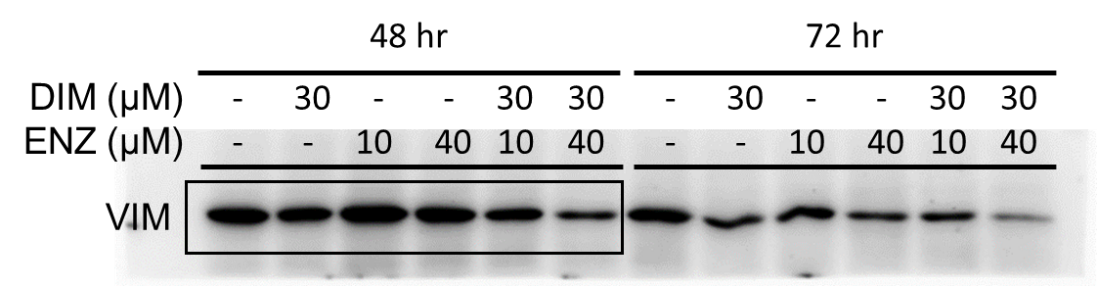


(D) β-actin_48 hr


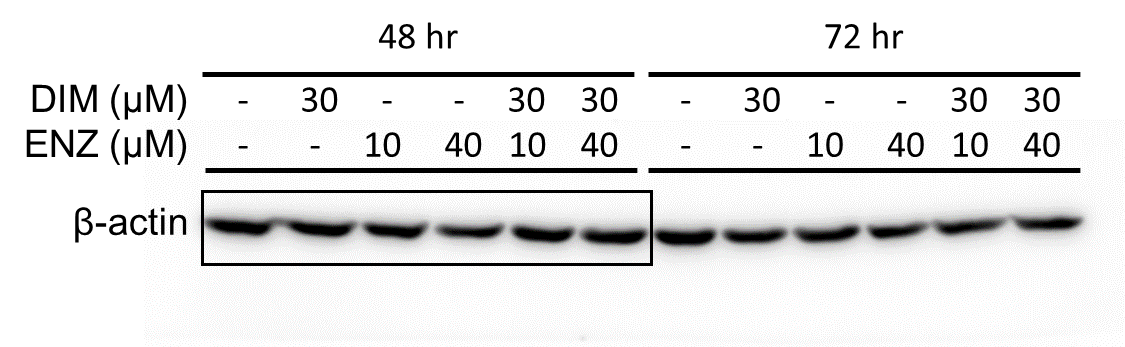
 (FIN, VIM)


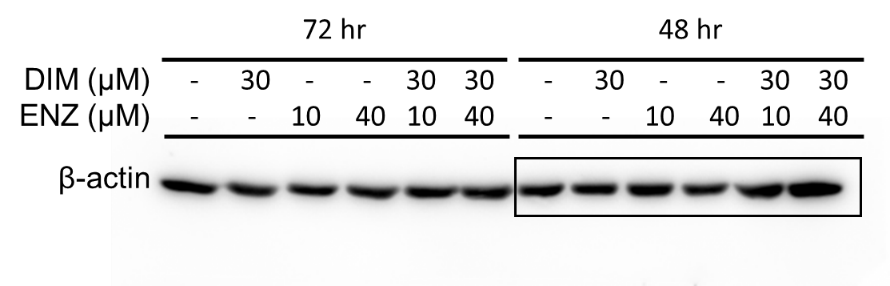
 (E-cad)

Supplementary Figure 4. Full-length western blot gels of Figure 9. Cropped gels are marked with a black box. FIN (A), E-cad (B), VIM (C), and β-actin (D). Image resolution = 600 dpi.

(A) FIN_72 hr


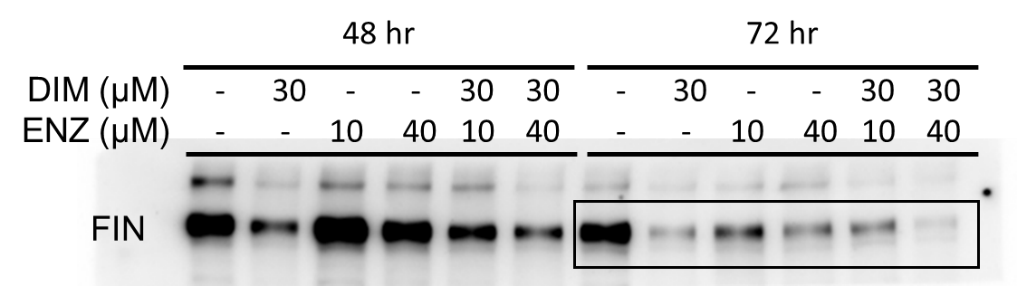


(B) E-cadherin_72 hr


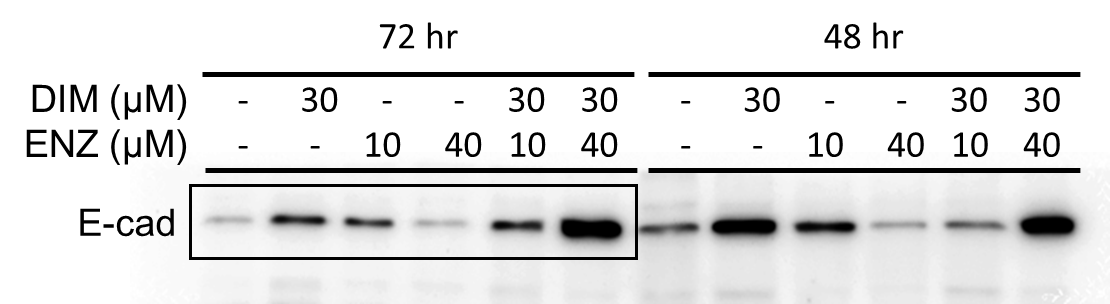


(C) VIM_72 hr


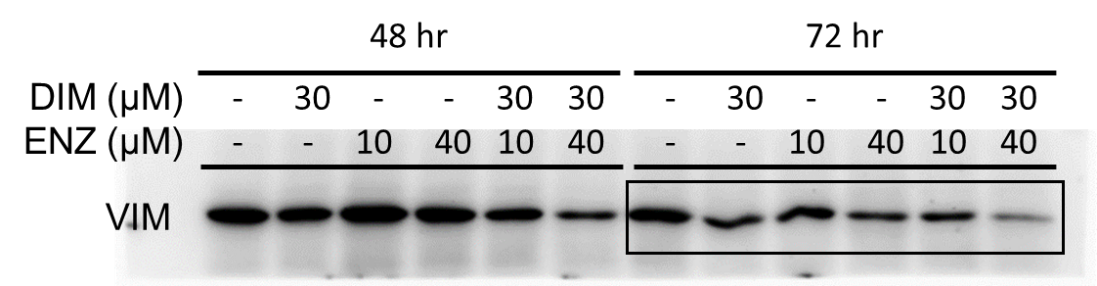


(D) β-actin_72 hr


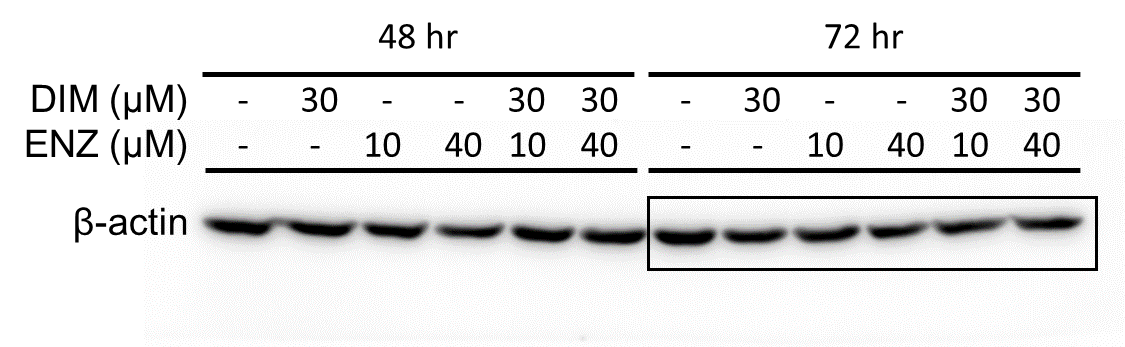
 (FIN, VIM)


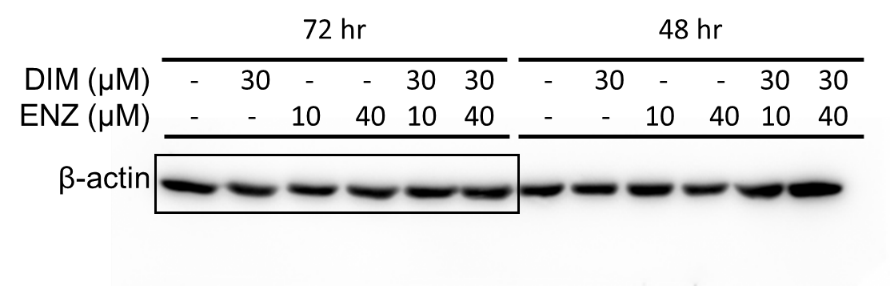
 (E-cad)

Supplementary Figure 5. Full-length western blot gels of Figure 10. Cropped gels are marked with a black box. FIN (A), E-cad (B), VIM (C), and β-actin (D). Image resolution = 600 dpi.

(A) PARP_48 hr


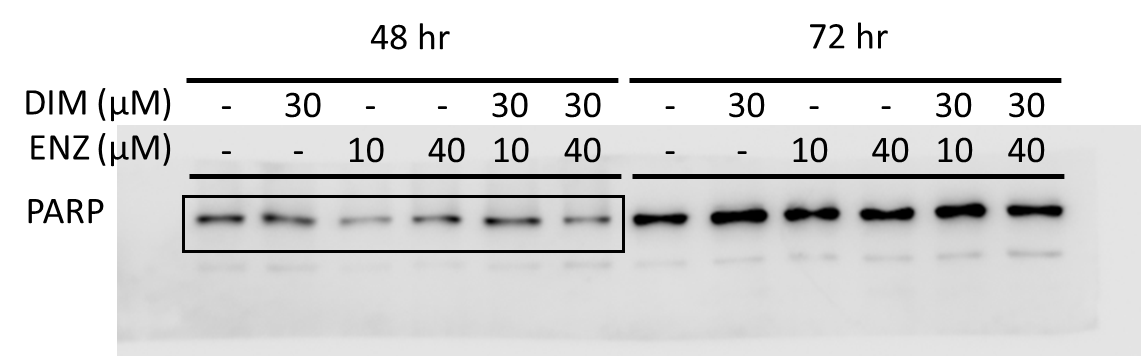


(B) Cleaved PARP_48 hr


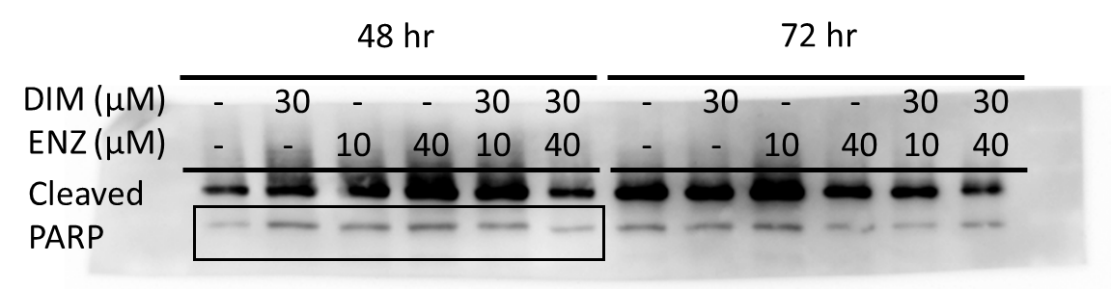


(C) Caspase 3_48 hr


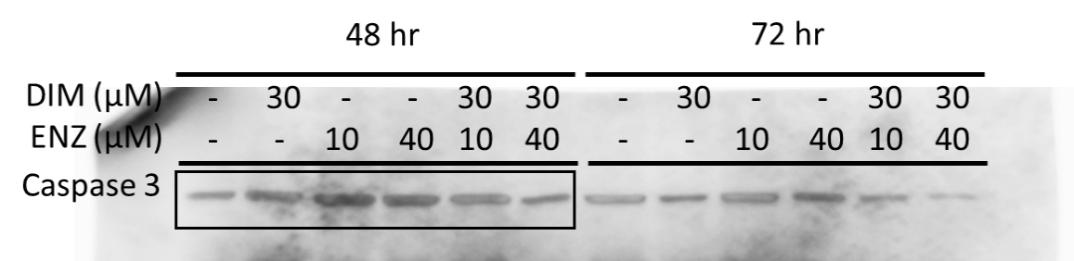


(D) Cleaved caspase 3_48 hr


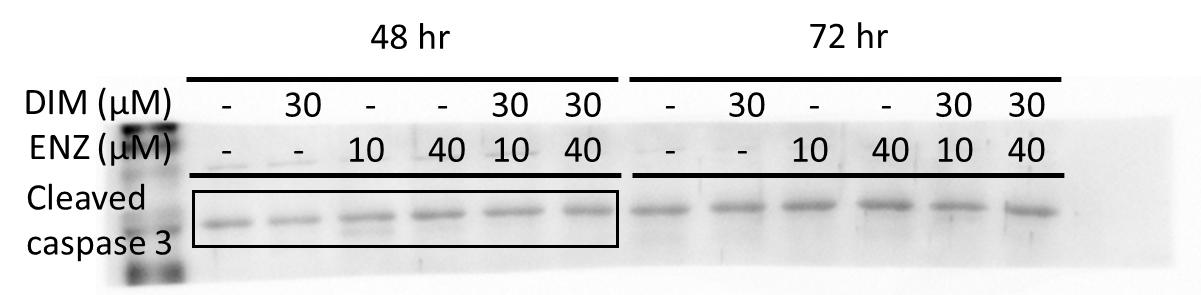


(E) β-actin_48 hr


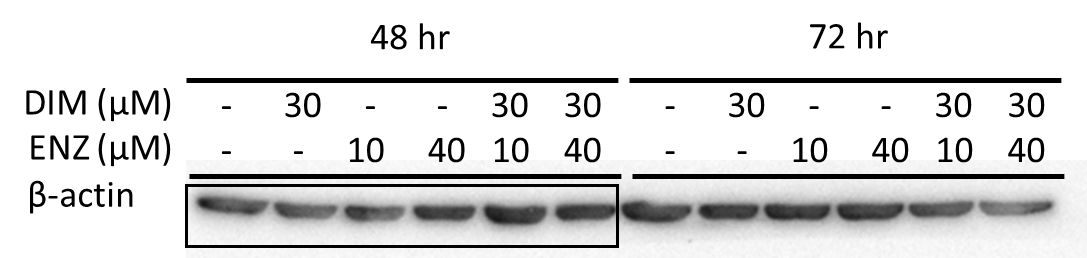


(PARP, Cleaved PARP, Caspase 3)


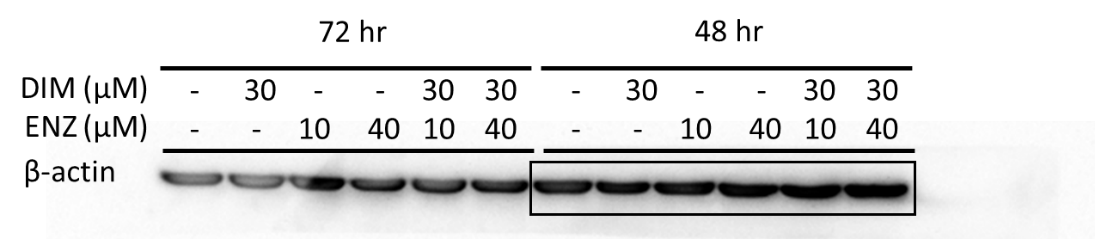


(Cleaved caspase 3)

Supplementary Figure 6. Full-length western blot gels of Figure 10. Cropped gels are marked with a black box. PARP (A), cleaved PARP (B), caspase 3 (C), cleaved caspase 3 (D), and β-actin (E). Image resolution = 600 dpi.

(A) PARP_72 hr


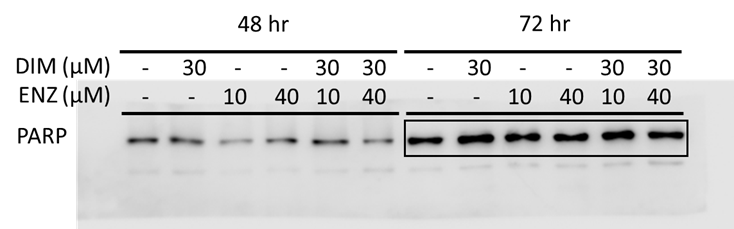


(B) Cleaved PARP_72 hr


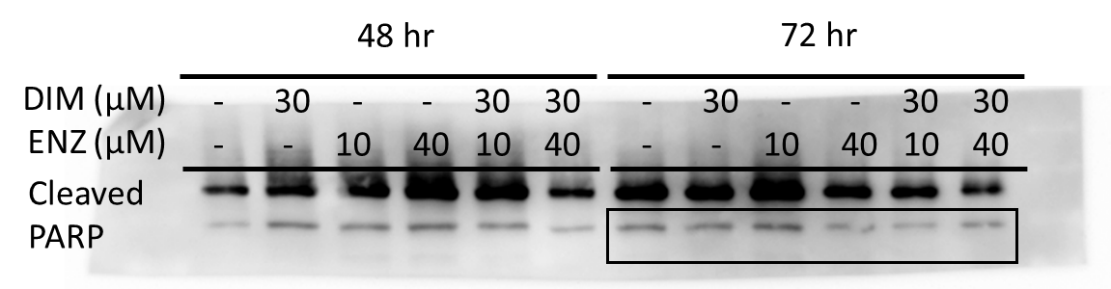


(C) Caspase 3_72 hr


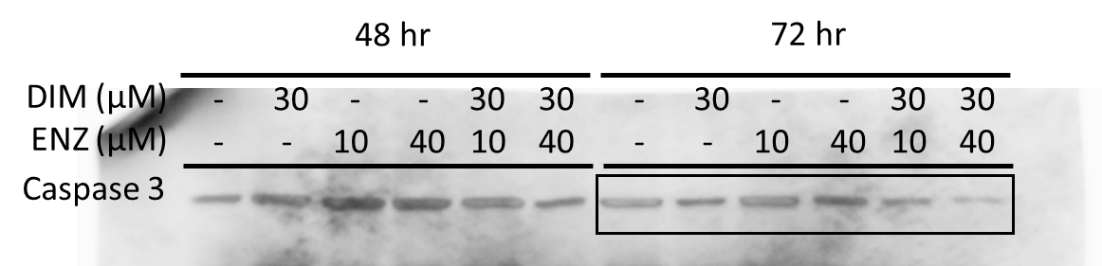


(D) Cleaved caspase 3_72 hr


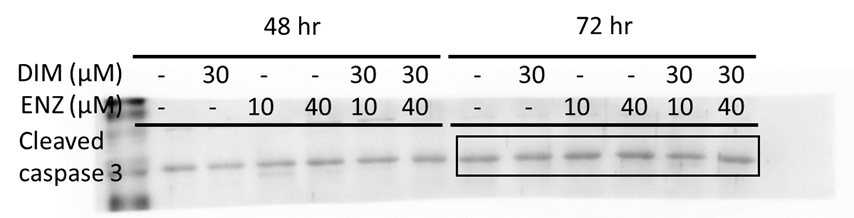


(E) β-actin_72 hr


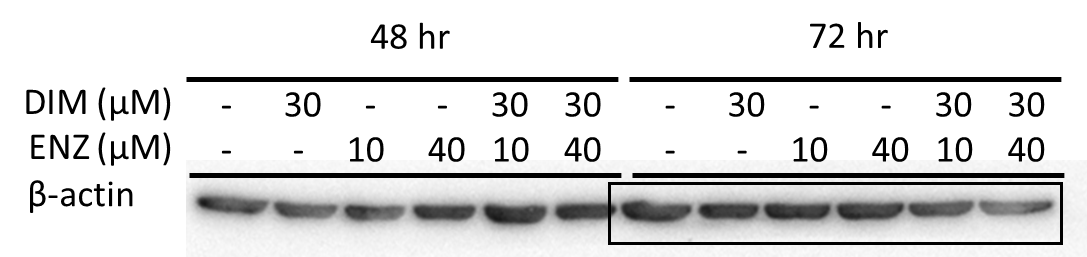


(PARP, Cleaved PARP, Caspase 3)


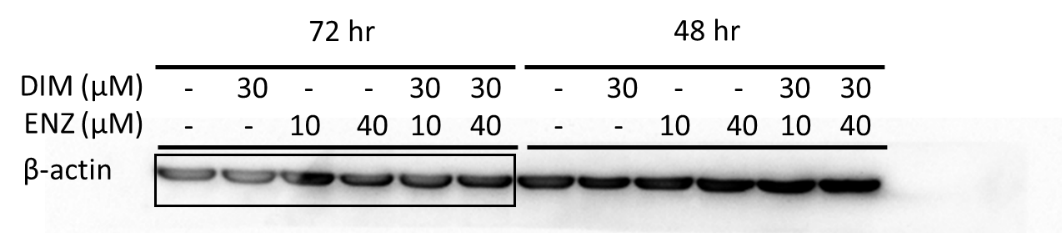


(Cleaved caspase 3)

Supplementary Figure 7. Full-length western blot gels of Figure 10. Cropped gels are marked with a black box. PARP (A), cleaved PARP (B), caspase 3 (C), cleaved caspase 3 (D), and β-actin (E). Image resolution = 600 dpi.
