# Supplementary material for: Regulation of carcinogenesis and mediation through Wnt/β-catenin signaling by 3,3′-diindolylmethane in an enzalutamide-resistant prostate cancer cell line
Source: Sci Rep. 2021 Jan 13;11:1239. doi: 10.1038/s41598-020-80519-3 (PMC7806813; doi:10.1038/s41598-020-80519-3)
Supplement: Supplementary file 2 — Supplementary Tables. [file 41598_2020_80519_MOESM2_ESM.docx]

**Supplementary Tables**

Table 1. Gene sequences of primers.

| Gene name | Forward primer | Reverse primer |
| --- | --- | --- |
| AR-FL | AGGATGCTCTACTTCGCCCC | ACTGGCTGTACATCCGGGAC |
| AR-v7 | CACATGTGGAAGCTGCAAGG | CAACCCGGAATTTTTCTCCCAG |
| E-cadherin  (CDH1) | CCCACCACGTACAAGGGTC | ATGCCATCGTTGTTCACTGGA |
| Fibronectin  (FN1) | GAAGCCGAGGTTTTAACTGC | ACCCACTCGGTAAGTGTTCC |
| Vimentin  (VIM) | GAACGCCAGATGCGTGAAATG | CCAGAGGGAGTGAATCCAGATTA |
| GAPDH | ACCCACTCCTCCACCTTTGACG | TCTCTTCCTCTTGTGCTCTTG |

Table 2. Antibodies used in the study.

| Name | Dilution ratio |
| --- | --- |
| Primary antibodies |  |
| AR (Santa Cruz, sc-7305) | 1:1000 |
| AR-v7 (Cell Signaling, 68492) | 1:1000 |
| APC (Santa Cruz, sc-9998) | 1:1000 |
| GSK3β (Cell Signaling, 12456) | 1:1000 |
| E-cadherin (Santa Cruz, sc-8426) | 1:1000 |
| Fibronectin (Santa Cruz, sc-8422) | 1:1000 |
| Vimentin (Santa Cruz, sc-6260) | 1:1000 |
| PARP (Cell Signaling, 3542) | 1:750 |
| Caspase-3 (Cell Signaling, 9662) | 1:750 |
| Cleaved Caspase-3 (Cell Signaling, 9664) | 1:750 |
| β-catenin (BD Biosciences, 610154) | 1:2000 |
| α-tubulin (GeneTex, GTX112141) | 1:1000 |
| β-actin (Sigma, A5316) | 1:10000 |
| Secondary antibodies |  |
| Goat anti-mouse IgG-HRP (Santa Cruz, sc-2005) | 1:5000 |
| Goat anti-rabbit IgG-HRP (Santa Cruz, sc-2004) | 1:4000 |
